# Mucosal IL-4R antagonist HIV vaccination with SOSIP-gp140 booster can induce high-quality cytotoxic CD4^+^/CD8^+^ T cells and humoral responses in macaques

**DOI:** 10.1038/s41598-020-79172-7

**Published:** 2020-12-16

**Authors:** Z. Li, M. Khanna, S. L. Grimley, P. Ellenberg, C. A. Gonelli, Wen Shi Lee, T. H. Amarasena, A. D. Kelleher, D. F. J. Purcell, S. J. Kent, C. Ranasinghe

**Affiliations:** 1 Molecular Mucosal Vaccine Immunology Group, Department of Immunology and Infectious Disease, The John Curtin School of Medical Research, The Australian National University, Canberra, ACT 2601 Australia; 2 Department of Microbiology, Immunology and Parasitology, Louisiana State University Health Sciences Center, New Orleans, LA 70112 USA; 3 Department of Microbiology and Immunology, Peter Doherty Institute for Infection and Immunity, University of Melbourne, Melbourne, VIC 3010 Australia; 4 Immunovirology and Pathogenesis Program, Kirby Institute, University of New South Wales, Sydney, NSW 2052 Australia

**Keywords:** Immunology, Vaccines, Adjuvants, Preclinical research

## Abstract

Inducing humoral, cellular and mucosal immunity is likely to improve the effectiveness of HIV-1 vaccine strategies. Here, we tested a vaccine regimen in pigtail macaques using an intranasal (i.n.) recombinant Fowl Pox Virus (FPV)-*gag pol env*-IL-4R antagonist prime, intramuscular (i.m.) recombinant Modified Vaccinia Ankara Virus (MVA)-*gag pol*-IL-4R antagonist boost followed by an i.m SOSIP-gp140 boost. The viral vector—expressed IL-4R antagonist transiently inhibited IL-4/IL-13 signalling at the vaccination site. The SOSIP booster not only induced gp140-specific IgG, ADCC (antibody-dependent cellular cytotoxicity) and some neutralisation activity, but also bolstered the HIV-specific cellular and humoral responses. Specifically, superior sustained systemic and mucosal HIV Gag-specific poly-functional/cytotoxic CD4^+^ and CD8^+^ T cells were detected with the IL-4R antagonist adjuvanted strategy compared to the unadjuvanted control. In the systemic compartment elevated Granzyme K expression was linked to CD4^+^ T cells, whilst Granzyme B/TIA-1 to CD8^+^ T cells. In contrast, the cytotoxic marker expression by mucosal CD4^+^ and CD8^+^ T cells differed according to the mucosal compartment. This vector-based mucosal IL-4R antagonist/SOSIP booster strategy, which promotes cytotoxic mucosal CD4^+^ T cells at the first line of defence, and cytotoxic CD4^+^ and CD8^+^ T cells plus functional antibodies in the blood, may prove valuable in combating mucosal infection with HIV-1 and warrants further investigation.

## Introduction

HIV/AIDS remains a global health problem with close to 38 million people living with the virus and 32 million lives taken by AIDS-related illnesses. Although around 64% of those living with HIV-1 have access to antiretroviral therapy, an effective HIV vaccine is thought to be the most cost-effective solution. Notwithstanding the last four decades of significant advances, development of an effective HIV vaccine strategy still remains a challenge. Unfortunately, many heterologous prime-boost vaccine strategies that have shown good promise in animal models, have yielded disappointing outcomes in human Phase I/II trials^1–5^. The RV144 canary poxvirus prime (ALVAC) alum gp120 protein booster trial was one of the first to show some promise in humans, with 31.2% protection associated with Env-specific antibodies and ADCC activity^6–8^. Disappointingly, phase IIb/III of this trial (HVTN 702), which used a bivalent gp120 MF59 adjuvant booster, was recently halted due to its inability to prevent HIV transmission in South African adults^9^. This failure has highlighted that an effective HIV vaccine strategy may need to induce mucosal immunity encompassing both the adaptive and humoral arms of the immune system to achieve effective protection.

HIV is first encountered at the genito-rectal mucosae, and early CD4 depletion occurs in the gut^10^. Many studies have now shown that mucosal administered vaccination can induce sustained higher avidity mucosal T cells with better protective efficacy compared to systemic delivery^11–16^, however there has been limited interest in taking a mucosal HIV vaccine towards larger human trials. When trying to unravel the mechanisms behind mucosal verses systemic delivery upon vaccine outcomes, we have recently found that innate lymphoid cells type 2 (ILC2)-derived IL-13 at the vaccination site plays an important role in shaping the downstream adaptive immune outcomes in mice^17–19^. Specifically, reduced IL-13 at the vaccination site associated with mucosal delivery leads to recruitment of conventional dendritic cells^18,20,21^, resulting in high avidity/poly-functional CD8^+^ T cells, with low or no IL-13 expression^13,22,23^. Moreover, we have also shown that although IL-13 is detrimental for high avidity T cell induction, the presence of low level of IL-13 is necessary for effective B cell immunity^24,25^. These observations have suggested that a vaccine strategy/adjuvant that can maintain low level of IL-13 at the vaccination site may prove useful in inducing a more effective/balanced T and B cell immune outcome against mucosal pathogens such as HIV.

We have shown that intranasal (i.n.) rFPV/intramuscular (i.m.) rMVA IL-4R antagonist adjuvanted vaccine strategy which transiently inhibit IL-4/IL-13 signalling via STAT6 at the vaccination site^17^ can induce not only robust mucosal and systemic high avidity/poly-functional cytotoxic HIV- or SIV-specific CD4^+^/CD8^+^ T cells but also Gag-specific B-cell immunity in both mice and non-human primates (NHP)^24,26^. This i.n./i.m. strategy induced protective immunity in a subset of pigtail macaques against a high dose SIV_mac251_ intra-rectal challenge. The protection observed was associated with poly-functional cytotoxic mucosal SIV-specific CD4^+^ T cells^26^. The importance of cytotoxic CD4^+^ T cells in protection against several viruses has been well-documented, including the recent respiratory syndrome coronavirus 2 (SARS-CoV2)^27–31^. A limitation of our previous approach, however, was the modest induction of functional Env-specific antibody responses, which may provide an additional line of defence against infection.

The monovalent gp120 Env used in the RV144 or HVTN702 vaccine trials do not mimic the gp160 Env trimers present on the surface of the virions. Stabilized gp140 trimers, called “SOSIP” trimers, show promise in the induction of more effective antibody responses^32^. These SOSIP Env trimers have not been studied for induction of a broader range of immune responses when used as a booster vaccine in the setting of a mucosal vector-based vaccine approach. In the current macaque immunogenicity study with HIV-1 vaccines, we integrated an i.m SOSIP-gp140 booster^33–37^ to our i.n. FPV-*gag pol env*-IL-4R antagonist prime/i.m. MVA-*gag pol*-IL-4R antagonist booster strategy. We assessed whether this approach could yield robust humoral and cellular HIV-specific immune responses in macaques.

## Results

### IL-4R antagonist vaccination strategy induced highly poly-functional HIV-1 Gag-specific systemic CD4^+^ and CD8^+^ T cell responses

**Figure 1:**
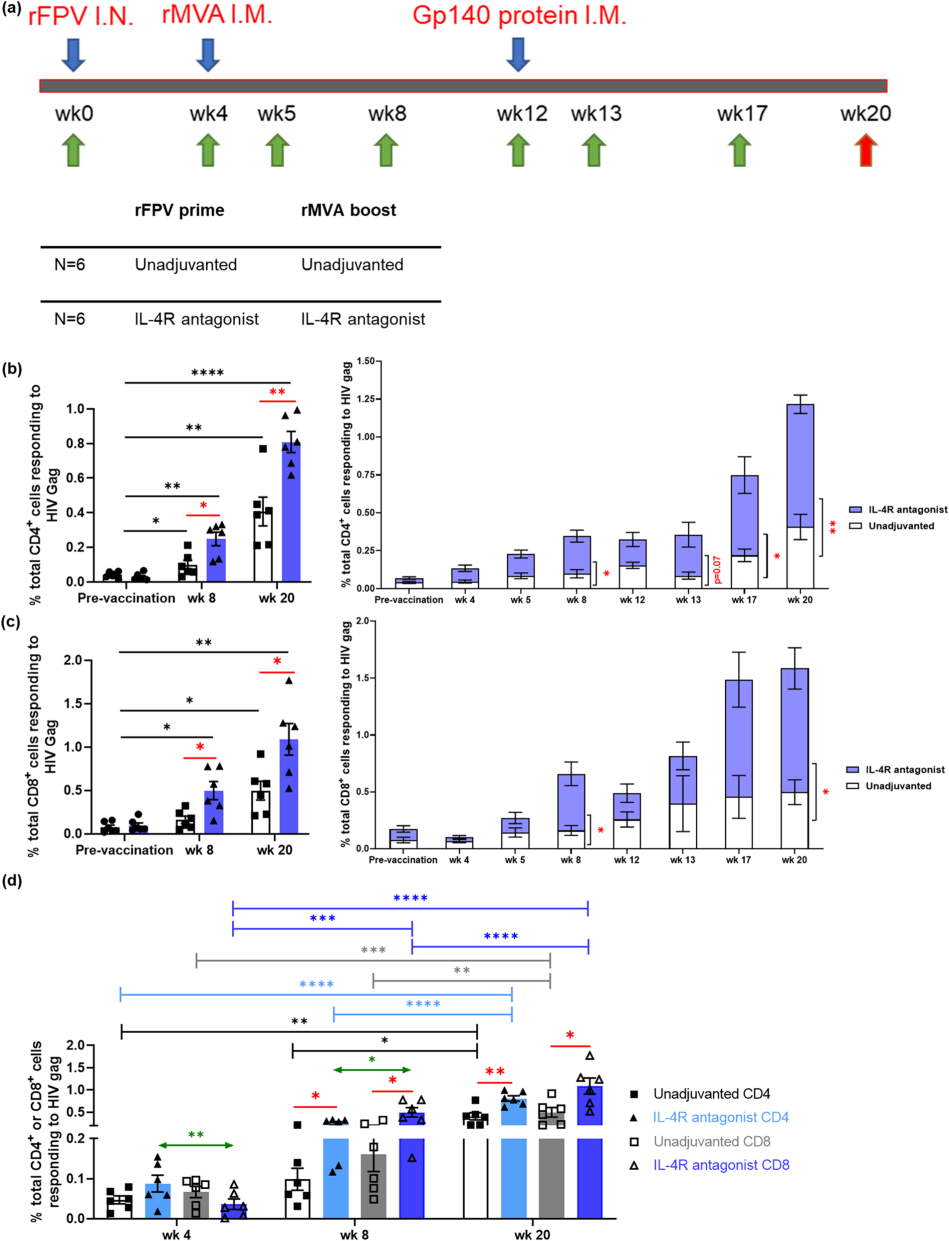
Evaluation of HIV Gag-specific cytokine expression by systemic CD4^+^ and CD8^+^ T cells. The animals were vaccinated, and samples were collected as per described in the timeline (**a**). Arrows indicate the following time points; blue—vaccination time, green—blood collection and red—autopsy. The HIV Gag-specific IFN-γ, TNF, IL-2, and CD107a expression by CD4^+^ and CD8^+^ T cells in fresh whole blood were measured using multi-colour flow cytometry, poly-functional and the summation or add-up analysis were performed as per described in "Methods", using gating strategy indicated in (S.Fig. 13). The bar graphs (left) represent comparison of total percentage of (**b**) CD4^+^ or (**c**) CD8^+^ T cells responding to HIV Gag (summation or add-up analysis) pre-vaccination, wk 8 (4 weeks post MVA), and wk 20. The stack bars (right) represent the total percentage of (**b**) CD4^+^ or (**c**) CD8^+^ T cells responding to HIV Gag at all time points tested. (**d**) Bar graph represents the comparison between CD4^+^ and CD8^+^ T cells responding to HIV Gag at three key time points (wk 4, wk 8, and wk 20). Blue bars represent IL-4R antagonist adjuvanted and white/grey bars represent unadjuvanted vaccination groups. The *p* values are denoted as: *ns—p ≥ *0.05, *—p < 0.05, **—p < 0.01. ***—p < 0.001, ****—p < 0.0001.

Two groups of animals (n = 6/group) were primed intranasally with 2 × 10^8^ PFU of FPV-HIV or FPV-HIV-IL-4R antagonist vaccines. Four weeks later, animals were boosted intramuscularly with 2 × 10^8^ PFU MVA-HIV or MVA-HIV-IL-4R antagonist vaccines, respectively. Eight weeks later, animals were boosted intramuscularly with 0.5 mg of SOSIP gp140 trimer together with AddaVax. Blood samples were collected over 20 weeks as indicated in the timeline (Fig. 1a). Effector and memory CD4^+^ and CD8^+^ T cell responses were evaluated using multi-colour flow cytometry. Both vaccine regimens induced Gag-specific IFN-γ, TNF, IL-2 and CD107a expression by CD4^+^ and CD8^+^ T cells (S.Fig. 1). In the context of Gag-specific CD4^+^ T cells, significantly elevated IL-2 expression was detected from 4 weeks post i.n. rFPV vaccination (wk4), whereas enhanced IFN-γ and TNF expression was detected 1 week post i.m. rMVA boost (wk5) (S.Fig. 1a–c) and enhanced CD107a expression 4 weeks post i.m. rMVA boost (wk8) (S.Fig. 1d). Similarly, elevated Gag-specific IFN-γ, IL-2, and CD107a expression by CD8^+^ T cells were detected 4 weeks post rMVA vaccination (wk8) (S.Fig. 11e, f & h) with elevated TNF expression detected at wk12 (S.Fig. 1f.). Remarkably, post i.m. AD8 SOSIP gp140 booster at 12 weeks, both vaccine groups showed significantly elevated HIV Gag-specific IFN-γ, TNF, IL-2 and CD107a expression by CD4^+^ and CD8^+^ T cells, which was sustained over time (S.Fig. 1 and Fig. 1). Compared to the unadjuvanted vaccine control, the IL-4R antagonist vaccinated group showed significantly elevated Gag-specific IFN-γ expression at wk17 (*p* = 0.037) & wk20 (*p* = 0.002) and IL-2 expression at wk13 (*p* = 0.047) by CD4^+^ T cells and CD107a expression by CD8^+^ T cells at 13–20 weeks (*p* = 0.03, 0.009, 0.04 respectively) (S.Fig.1 and Fig. 1).

**Figure 2:**
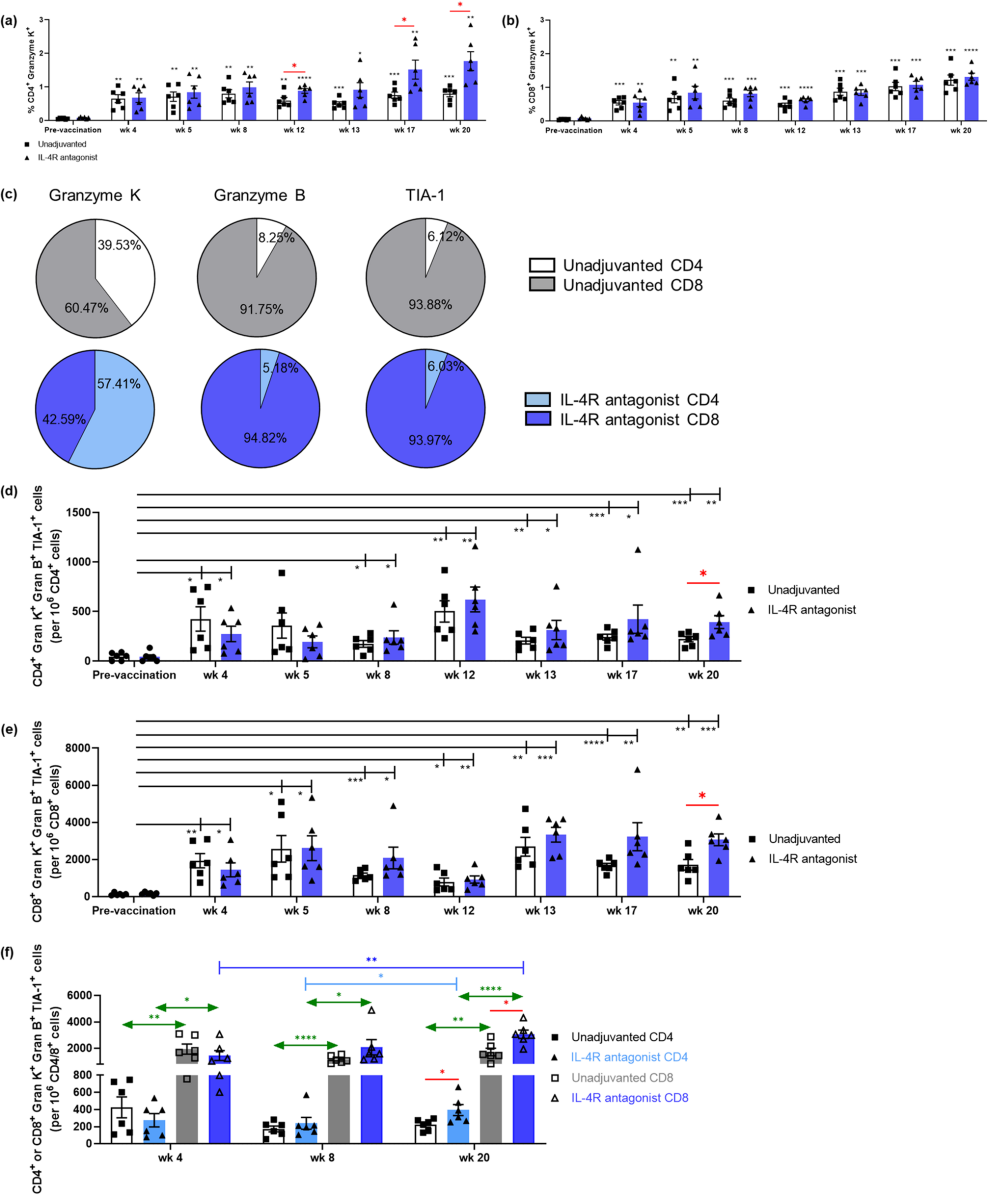
Evaluation of Granzyme B, K, and TIA-1 expression by systemic CD4^+^ and CD8^+^ T cells. The total cytotoxic T cell potential pre- and post-vaccination was assessed as per described in "Methods", with no peptide stimulation. The bar graphs represent percentage of (**a**) CD4^+^ T cell and (**b**) CD8^+^ T cell expressing Granzyme K at all time points tested. The black asterisks on top of each bar indicate the relative statistical significance compared to pre-vaccination, and the red asterisks indicate the statistical significance between the two vaccination groups. (**c**) Pie charts represent proportion of Granzyme K, B and TIA-1 expression in CD4^+^ or CD8^+^ cells at wk 20. (**d**, **e**) Bar graphs represent the number of triple positive (**d**) CD4^+^ and (**e**) CD8^+^ T cells, back calculated to 1 × 10^6^ T cells. (**f**) Bar graph represents the comparison of the number of triple positive CD4^+^ and CD8^+^ T cells in both vaccination groups at three key intervals (wk 4, wk 8, and wk 20). Blue bars represent IL-4R antagonist adjuvanted and white/grey bars represent unadjuvanted vaccination groups. The *p* values are denoted as: *ns—p ≥ *0.05, *—p < 0.05, **—p < 0.01. ***—p < 0.001, ****—p < 0.0001.

To assess the magnitude of the total cellular immune response to Gag, we summated the proportion of CD4^+^ or CD8^+^ T cells expressing IFN-γ, TNF, IL-2 or CD107a expression as described in "Methods" (S.Fig. 13). In both vaccine groups tested, from wk5 to wk20, the proportion of total CD4^+^ cells responding to HIV Gag were elevated compared to baseline (Fig. 1b, S.Fig. 2). The IL-4R antagonist vaccine also showed elevated Gag-specific CD4^+^ T cell responses from 4 weeks post i.n. rFPV compared to pre-vaccination baseline (S.Fig. 2a). The i.m. AD8 SOSIP gp140 booster significantly enhanced the proportion of total CD4^+^ T cells responding to HIV Gag from 17 to 20 weeks (Fig. 1b, S.Fig. 2a). Moreover, the IL-4R antagonist adjuvanted vaccine strategy generated a significantly greater proportion of total CD4^+^ T cell responding to HIV Gag at wk8 (4 weeks post rMVA vaccination, *p* = 0.013), wk17 (5 weeks post gp140 protein booster, *p* = 0.048) and wk20 (8 weeks post gp140 protein booster, *p* = 0.003) (Fig. 1b, S.Fig. 2a) compared to the unadjuvanted vaccine regimen. For CD8^+^ T cell responses, a significantly greater proportion of cells responding to HIV Gag were detected with IL-4R antagonist adjuvanted vaccinated group compared to the unadjuvanted control at wk8 (*p* = 0.021) and wk20 (*p* = 0.023) (Fig. 1c, S.Fig. 2c).

We next evaluated whether the vaccine regimens primarily induced CD4^+^ or CD8^+^ T cell responses. The recipients of the unadjuvanted vaccine strategy showed no significant difference in the magnitude of the total Gag-specific CD4 or CD8 T cell responses (Fig. 1d). However, the IL-4R antagonist vaccinated group showed significantly elevated Gag-specific CD4^+^ compared to CD8^+^ T cells at wk4 (*p* = 0.003), which was reversed following the rMVA booster (wk8) (*p* = 0.039) (Fig. 1d). The IL-4R antagonist also showed a greater proportion of Gag-specific CD4^+^ and CD8^+^ T cells compared to unadjuvanted control at week 20, the end of the study (*p* = 0.003, 0.023 respectively) (Fig. 1d).

**Figure 3:**
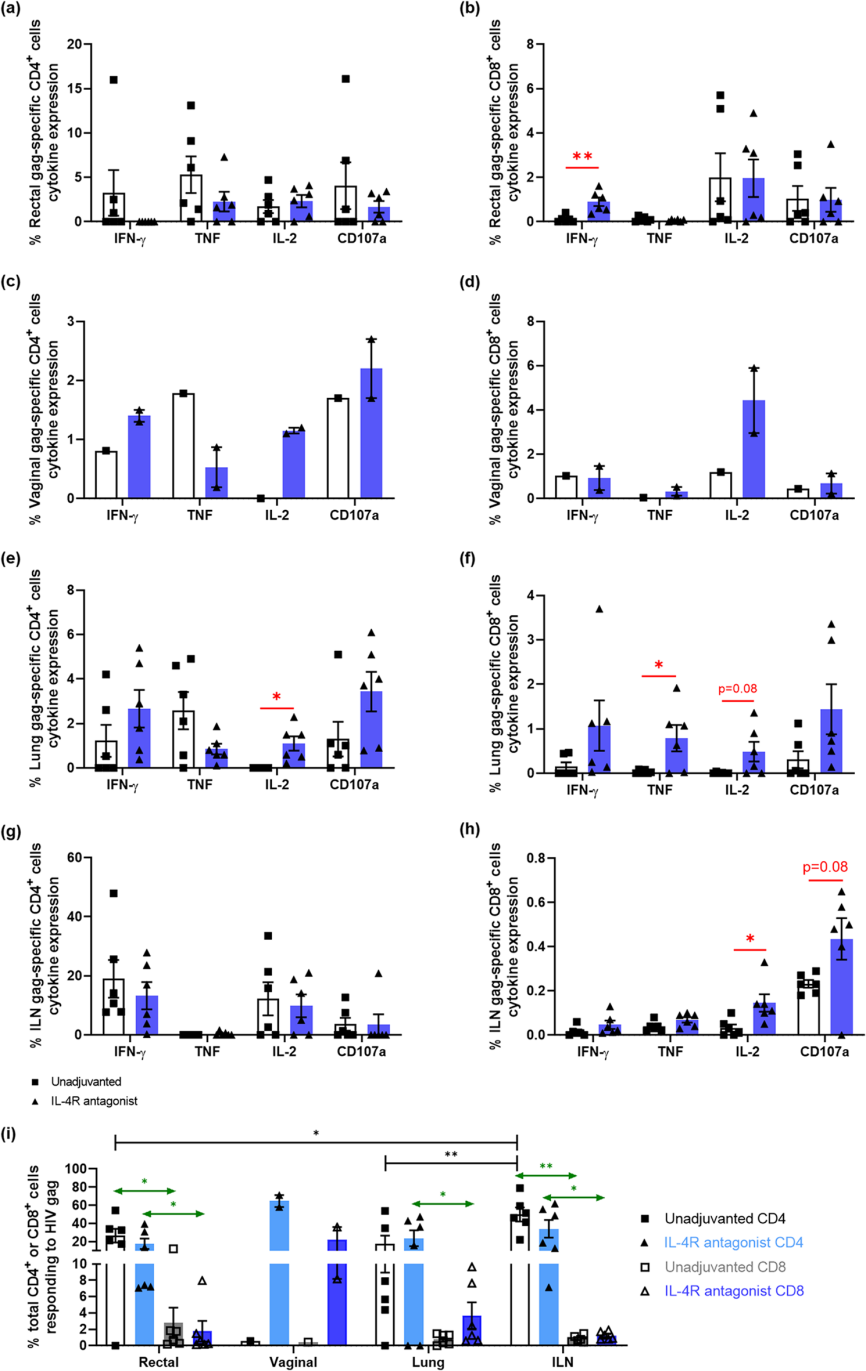
Evaluation of HIV-Gag specific mucosal CD4^+^ and CD8^+^ T cell cytokine expression profiles. The animals were ethically euthanized at wk 20 and rectal, vaginal, lung and ILN tissues were collected. Single cell suspensions were prepared, and multi-colour intracellular flow cytometry analysis was performed to evaluate vaccine-specific cytokine and CD107a expression by CD4^+^ and CD8^+^ T cells as per described in "Methods". The bar charts represent the percentage of CD4^+^ (left) and CD8^+^ (right) T cells expressing IFN-γ, TNF, IL-2, and CD107a in (**a**, **b**—rectal; **c**, **d**—vaginal; **e**, **f**—lung; **g**, **h**—ILN) mucosal tissue compartments. (i) Bar graph represents the summation or add-up analysis of IFN-γ, TNF, IL-2, and CD107a expression by CD4^+^ and CD8^+^ T cells in different tissue compartments. In this study, blue bars represent IL-4R antagonist adjuvanted and white/grey bars represent unadjuvanted vaccination groups. The *p* values are denoted as: *ns—p ≥ *0.05, *—p < 0.05, **—p < 0.01. ***—p < 0.001, ****—p < 0.0001.

**Figure 4:**
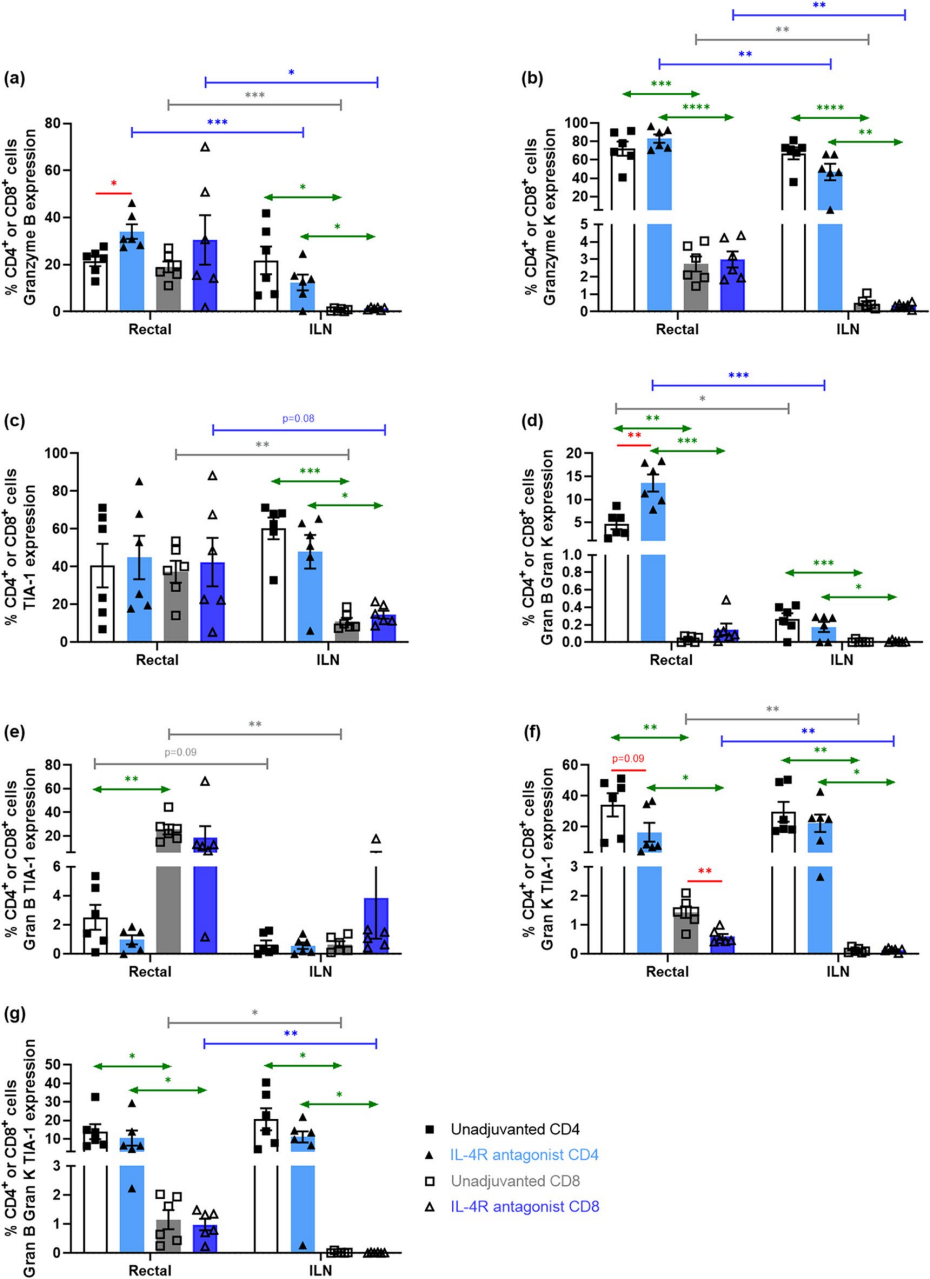
Evaluation of Granzyme B, K, and TIA-1 expression by mucosal CD4^+^ and CD8^+^ T cells. Single cell suspensions were prepared, and multi-colour intracellular flow cytometry analysis was as per described in "Methods". Due to the limited cell yields, expression of Granzyme B, K, and TIA-1 by CD4^+^ and CD8^+^ T cells were only evaluated in rectal tissue and ILN. The bar charts indicate percentage of CD4^+^ and CD8^+^ T cells expressing (**a**) Granzyme B, (**b**) Granzyme K, (**c**) TIA-1, and double positive for (**d**) Granzyme B and K, (**e**) Granzyme B and TIA-1, (**f**) Granzyme K and TIA-1, as well as (**g**) triple positive T cells in both rectal tissue and ILN. The blue bars represent IL-4R antagonist adjuvanted and white/grey bars represent unadjuvanted vaccination groups. The *p* values are denoted as: *ns—p ≥ *0.05, *—p < 0.05, **—p < 0.01. ***—p < 0.001, ****—p < 0.0001.

The rFPV and rMVA vaccines also expressed HIV-1 Pol, another relatively conserved target for T cell immunity. The animals that received the IL-4R antagonist vaccine strategy also displayed significantly elevated HIV Pol-specific CD4^+^ T cell expressing IFN-γ at wk8 and wk17 (*p* = 0.022, 0.027 respectively) and IL-2 at wk13 (*p* = 0.041) compared to the unadjuvanted control. Moreover, HIV Pol-specific CD107a expression was found to be significantly elevated in both CD4^+^ at wk20 (*p* = 0.044) and CD8^+^ T cells at wk17 (*p* = 0.045) (S.Fig. 3). No significant difference in the total summation analysis of cytokine/cytotoxic T cells to HIV Pol were detected between the two vaccine groups at all time points tested (S.Fig. 4).

### IL-4R antagonist adjuvanted vaccination induced cytotoxic systemic CD4^+^ and CD8^+^ T cells expressing Granzyme K/B, and TIA-1

To further evaluate the cytotoxicity of systemic the vaccine-induced CD4^+^ and CD8^+^ T cells, the expression trends of Granzyme K, Granzyme B and TIA-1 (cytotoxic granule-associated RNA binding protein) were monitored over time, from baseline to 20 weeks (with no peptide stimulation). Unlike Granzyme B and TIA-1 expression, significantly elevated Granzyme K expression was detected in both CD4^+^ and CD8^+^ T cells from week 4 onwards (4 weeks post i.n. rFPV vaccination), and this expression was significantly increased over time (Fig. 2a, b). In the context of the IL-4R antagonist vaccinated group, significantly elevated Granzyme K expression by CD4^+^ T cells were detected at wk12 (*p* = 0.032), wk17 (*p* = 0.042), and wk20 (*p* = 0.016) compared to the unadjuvanted control group, unlike CD8^+^ T cells (Fig. 2a, b). In contrast to Granzyme K, the Granzyme B and TIA-1 were predominately expressed by CD8^+^ T cells (Fig. 2c).

**Figure 5:**
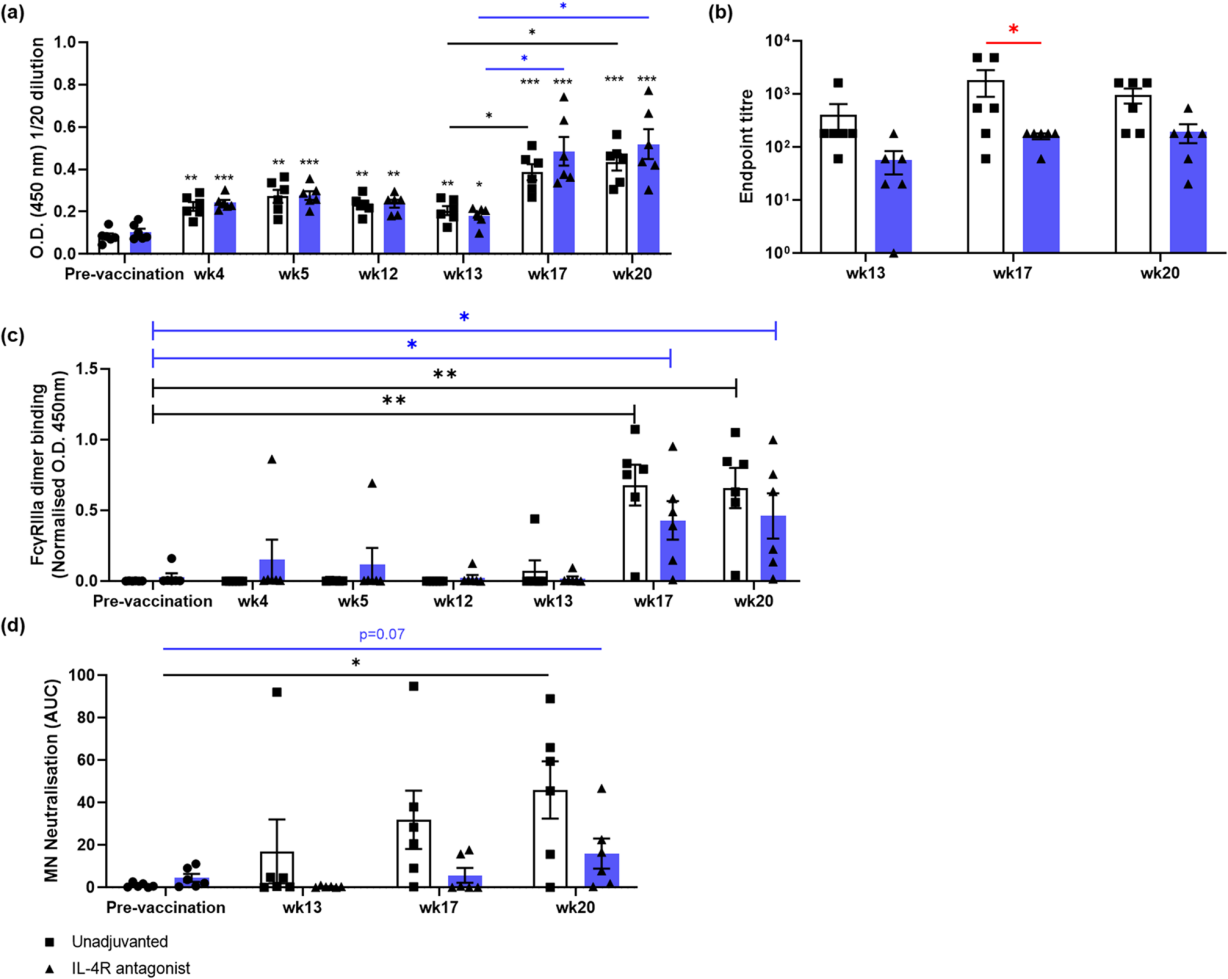
Assessment of HIV gp140 Env-specific total IgG and neutralisation antibody ADCC responses. ELISA was performed to assess (**a**) HIV gp140 Env-specific total IgG in the plasma as per described in "Methods". The black asterisks without a line on top of each bar indicate the relative statistical significance compared to pre-vaccination. (**b**) Bar graph represents the relevant endpoint titres, calculated as per described in "Methods" (**c**) Bar graph represents gp140-specific ADCC activity at wk4 to wk 20 compared to pre-vaccination time point. (**d**) Bar graph represents MN neutralisation represented as Area Under the Curve (AUC) at wk13, wk17 and wk20, compared to the pre-vaccination time point. The blue bars represent IL-4R antagonist adjuvanted and white bars represent unadjuvanted vaccination groups. The *p* values are denoted as: *ns—p ≥ *0.05, *—p < 0.05, **—p < 0.01. ***—p < 0.001, ****—p < 0.0001.

The expression of multiple cytotoxicity markers may reflect cells with high cytotoxic potential. When a poly-functional analysis of the three cytotoxic markers was performed, a gradual increase in Granzyme K, Granzyme B, and TIA-1 triple positive CD4^+^ and CD8^+^ T cells was detected from 4 weeks post rFPV vaccination to 20 weeks with both vaccine regimens (Fig. 2d, e, S.Fig. 5). However, by 20 weeks the IL-4R antagonist vaccination showed greater Granzyme K, Granzyme B and TIA-1 triple positive CD4^+^ T cells as well as CD8^+^ T cells compared to the unadjuvanted control (*p* = 0.043, 0.011 respectively) (Fig. 2d, e). Specifically, the number of Granzyme K^+^ Granzyme B^+^ TIA-1^+^ CD4^+^ T cells were greater at wk20 compared to wk8 (*p* = 0.015), whereas in the context of triple positive CD8^+^ T cells was much greater at wk20 compared to wk4 (*p* = 0.0011) (Fig. 2f). As expected, the cytotoxic potential of CD8^+^ T cells was greater than CD4^+^ T cells as the number of Granzyme K^+^ Granzyme B^+^ TIA-1^+^ CD8^+^ T cells were significantly higher than for CD4^+^ T cells (*p* < 0.05–0.0001) (Fig. 2f).

### Both vaccine regimens induced elevated mucosal Gag-specific poly-functional/cytotoxic CD4^+^ T cells compared to CD8^+^ T cells

Our priming vaccine was administered i.n. and has been previously been associated with mucosal immune responses. Our previous macaque work, however, was limited to autopsy tissues after SIV challenge where the infection influenced the mucosal immunity. This HIV study allowed the monitoring of animals until autopsy post vaccination for a more in-depth analysis of mucosal immunity. HIV Gag-specific cytokine and CD107a expressing CD4^+^ and CD8^+^ T cells were detected in rectal, vaginal, lung tissues and iliac lymph nodes (ILN) at the autopsy (wk20) (Fig. 3a–h). The 2 vaccine regimens showed no significant difference in Gag-specific IFN-γ, TNF, IL-2 or CD107a expression in CD4^+^ T cells in each compartment (Fig. 3a, c, e, g) with the exception of elevated IL-2 expression in lung post IL-4R antagonist adjuvant vaccination (*p* = 0.019) (Fig. 3e). Overall, compared to the mucosal tissues, a greater percentage of Gag-specific ILN CD4^+^ T cells were found to be IFN-γ and IL-2 positive (Fig. 3g). In the context of Gag-specific CD8^+^ T cells, animals vaccinated with the IL-4R antagonist displayed significantly greater number of Gag-specific rectal CD8^+^ IFN-γ^+^ T cells compared to the unadjuvanted control (*p* = 0.008) (Fig. 3b). In the vaginal compartment, due to the limited number of female animals in each group (1 unadjuvanted and 2 adjuvanted), meaningful statistical analysis could not be performed. However, IL-4R antagonist vaccine showed a trend where much a greater Gag-specific CD4^+^ T cells expressing IFN-γ, IL-2, and CD107a (Fig. 3c), and CD8^+^ T cells expressing IL-2 were detected compared to the control (Fig. 3d). In the lung tissue, the IL-4R antagonist vaccines showed elevated Gag-specific CD8^+^ TNF^+^ (*p* = 0.049) (Fig. 3f). Similarly, in ILN, IL-4R antagonist vaccines induced elevated Gag-specific CD8^+^ IL-2^+^ T cells (*p* = 0.034) (Fig. 3h).

To obtain an overall view of the vaccine immunogenicity, as above for the blood T cell analyses, we summated all mucosal Gag-specific cells. Both vaccines showed significantly greater total Gag-specific CD4^+^ T cells in the rectal, lung and ILN compartments than CD8^+^ T cells (rectal *p* = 0.013* and p* = 0.006; lung *p* = 0.048;* ILN p* = 0.005* and p* = 0.02 control and IL-4R adjuvanted vaccines respectively) (Fig. 3i). Vaginal tissues the CD4^+^ and CD8 ^+^ T cells were greater in IL-4R adjuvanted group compared to the control albeit with limited numbers of animals per group (Fig. 3i).

### Mucosal CD4^+^ and CD8^+^ T cells show compartment-specific Granzyme B, K and TIA-1 expression profiles

**Figure 6:**
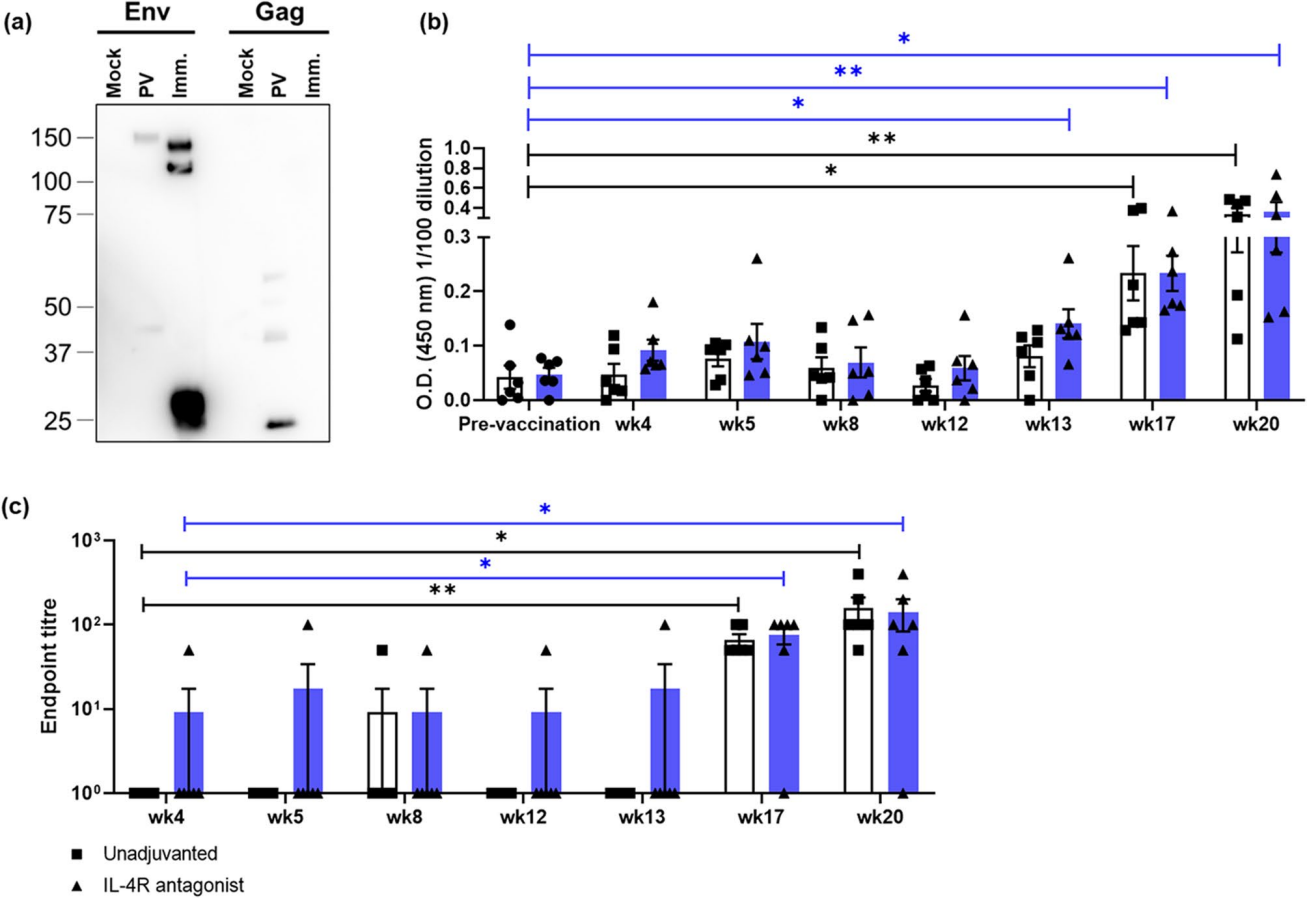
Evaluation of HIV Gag-specific humoral responses. (**a**) The presence or absence of HIV-Env (left) and HIV-Gag (right) in the SOSIP-gp140 protein booster was assessed using a SDS-PAGE and Western blot analysis probing for the respective proteins. (**b**) Bar graphs represent HIV p24 Gag-specific total IgG levels in plasma measured by ELISA, and (**c**) the respective endpoint titres, calculated as per described in "Methods". The blue bars represent IL-4R antagonist adjuvanted and white bars represent unadjuvanted vaccination groups. The *p* values are denoted as: *ns—p ≥ *0.05, *—p < 0.05, **—p < 0.01. ***—p < 0.001, ****—p < 0.0001.

At autopsy after vaccination, both vaccines showed different Granzyme B, K and TIA-1 expression profiles in rectal and ILN compartments (Fig. 4a–g). Note that due to limited cell yields, the expression of cytotoxic molecules was only examined in rectal tissue and ILN. The IL-4R antagonist vaccine group showed significantly elevated rectal CD4^+^ T cells expressing Granzyme B (*p* = 0.013) unlike ILN (Fig. 4a, d). Also, the Granzyme B expression in IL-4R antagonist vaccinated rectal CD4^+^ and CD8^+^ T cells was found to much greater than ILN (*p* = 0.0008, 0.0386 respectively) (Fig. 4a). Interestingly, much greater proportion of rectal and ILN CD4^+^ T cells expressed granzyme K than granzyme B or TIA-1 (S.Fig. 6). Furthermore, even though there was no difference between the two vaccine groups, percentage of CD4^+^ GnzK^+^ was much greater than CD8^+^ GnzK^+^ in both rectal and ILN compartments (rectal *p* = 0.0003, 0.00001*;* ILN *p* = 0.0001, 0.0036 respectively) (Fig. 4b). Similar to granzyme B, there was also a greater proportion of IL-4R antagonist vaccinated rectal CD4^+^ and CD8^+^ T cells expressing granzyme K compared to ILN (*p* = 0.008, 0.002 respectively) (Fig. 4b). In the context of TIA-1 although in rectal tissue no differences were observed between the vaccines or the T cell subtype (Fig. 4c), the TIA-1 expression was much greater in CD4^+^ than CD8^+^ T cells in ILN (Control—*p* = 0.0004 IL-4R antagonist—*p* = 0.0269) (Fig. 4c). Also, expression of TIA-1 was greater in rectal CD8^+^ T cells than ILN (Control—*p* = 0.005 IL-4R antagonist—*p* = 0.08) (Fig. 4c).

The expression of both granzyme B and K was significantly higher in the IL-4R antagonist vaccinated rectal CD4^+^ T cells compared to the control vaccination (*p* = 0.003) or rectal CD8^+^ T cells (*p* = 0.0008) (Fig. 4d). Interestingly, this expression was limited to rectal CD4^+^ T cells (Fig. 4d). In contrast even though both vaccine strategies showed similar granzyme B and TIA-1 double positive profile, expression was significantly higher in rectal CD8^+^ T cells than CD4^+^ T cells (Control—*p* = 0.0033) (Fig. 4e). Moreover, granzyme B and TIA-1 double positive T cells were much lower in ILN compared to rectal tissue (Fig. 4e). IL-4R antagonist vaccination group showed significantly reduced Granzyme K and TIA-1 double positive rectal CD4^+^ and CD8^+^ T cells compared to the control vaccination (*p* = 0.09, 0.006 respectively) (Fig. 4f). Overall, much lower Granzyme K and TIA-1 double positive CD8^+^ T cells were detected in rectal tissue and ILN (*rectal p* = 0.008, 0.051*; ILN p* = 0.006, 0.012 respectively) (Fig. 4f). Interestingly, significantly higher proportion of rectal and ILN CD4^+^ T cells were also found to be triple positive for Granzyme K, Granzyme B, TIA-1 compared to CD8^+^ T cells (*rectal p* = 0.023, 0.048;* ILN p* = 0.018, 0.015 respectively) (Fig. 4g) and also the triple positive rectal CD8^+^ T cells were greater than ILN CD8^+^ T cells (*p* = 0.018, 0.005 respectively) (Fig. 4g).

### Gp140-specific IgG, ADCC activity and marginal MN-specific neutralisation were detected following the AD8 SOSIP gp140 booster

**Figure 7:**
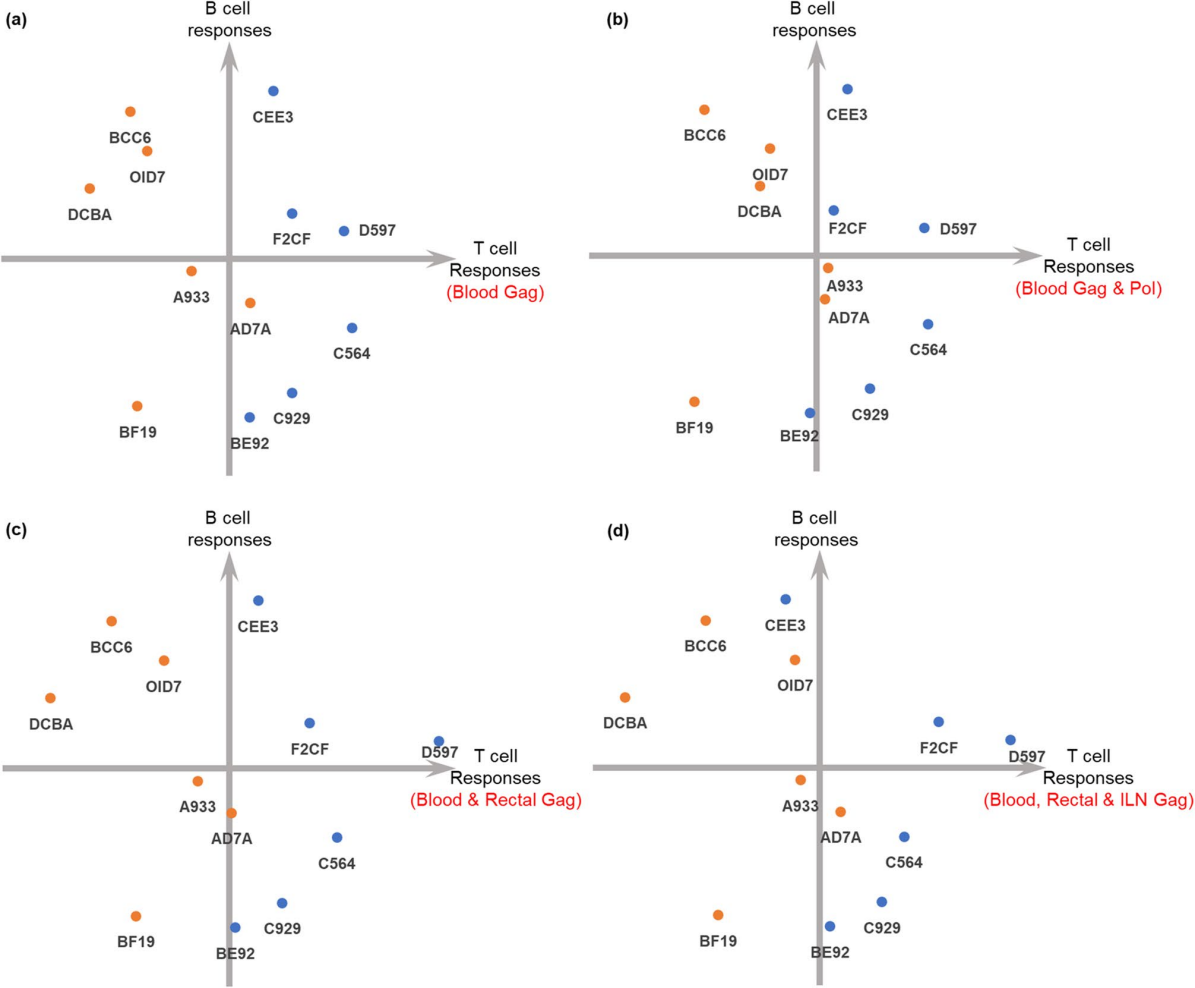
Summary quadrant plots of the vaccine trial outcome. The data from this vaccine trial was graphed in the format of quadrant plots to view the overall impact of the two vaccination strategies. Each dot represented an individual animal colour coded as, IL-4R antagonist adjuvanted vaccination (blue) and unadjuvanted control vaccination (orange). Next to each dot, the animal number is also included. Unpaired two-tail T test was performed to evaluate the statistical significance between the two vaccination groups. Horizontal axis represent T cell responses and vertical axis represent B cell responses.

In this study the i.n. rFPV priming vaccine expressed Env and we administered a gp140 protein boost at week 12. HIV gp140-specific IgG antibody responses were detected by ELISA at 4 weeks, following i.n. rFPV prime vaccination (*p* = 0.006* unadjuvanted control and *0.0002 *IL-4R antagonist vaccine strategy compared to pre-vaccination*)*,* and these responses were maintained at a similar level (O.D. ~ 0.3) until the AD8 SOSIP gp140 protein boost (Fig. 5a). Five weeks post AD8 SOSIP gp140 booster (wk17) both vaccination groups showed enhanced HIV gp140-specific IgG antibody responses compared to pre-protein booster (13w) (O.D. 450 ~ 0.4–0.5, *p* = 0.02* unadjuvanted and *0.025* IL-4R antagonist adjuvanted*) (Fig. 5a, S.Fig. 7). No significant differences were detected between the two vaccine groups at 20 weeks (Fig. 5a). In the context of endpoint titres, at 17 weeks the animals immunized with the unadjuvanted vaccine showed some increase in the gp140-specific IgG response compared to the IL-4R antagonist adjuvanted (*p* = 0.03) (Fig. 5b), which was not followed through to 20 weeks.

Fc-functional antibodies can play a role in protective immunity to HIV^7^ and we therefore next analysed the ability of Env antibodies in plasma to bind to a FcγRIIIa dimer, a surrogate of HIV-specific ADCC activity^38^. When FcγRIIIa dimer antibody responses against both gp140 and gp120 were assessed, most animals displayed gp140-specific activity by 5 weeks post AD8 SOSIP Gp140 booster (wk17) and which was maintained until the end of the trial (wk20) (Fig. 5c, S.Fig. 8a). No significant differences in FcγRIIIa dimer binding antibody responses were observed between the two vaccine groups (Fig. 5c). No gp120-specific ADCC activity was detected at any time points tested (S.Fig. 8b, c). Next, neutralising activity was tested against three different HIV-1 pseudoviruses (ADA, JRCSF,MN), as well as pseudovirus expressing the Env of Murine Leukemia virus (MuLV) as a negative control (S.Fig. 9a), as per described in "Methods". At wk13, wk17, and wk20, only the common MN pseudovirus showed susceptibility to neutralisation by immunised NHP plasma samples, but not the ADA and/or JRCSF strains. Animals in both vaccination groups developed weak, but some degree of MN neutralisation by wk20 (Fig. 5d, S.Fig. 9a, b). It is noteworthy that, although conformationally stabilised SOSIP gp140 proteins have reported to induce tier 1 and autologous tier 2 neutralising antibody responses, they have typically failed to induce broad neutralising antibody responses^39,40^.

Similar to the significant enhancement of Gag-specific CD4^+^ and CD8^+^ T cells responses (Fig. 1, S.Fig. 1, 2), even though the gp140 protein booster AD8 SOSIP gp140 preparation did not to contain any Gag protein, which was confirmed by the Western blot analysis (Fig. 6a, S.Fig. 10), significantly enhanced HIV p24 Gag-specific IgG antibody responses were also observed. IL-4R antagonist vaccinated group (*wk13 p* = 0.013,* wk17 p* = *0*.0013* and wk20 p* = 0.018 respectively), unadjuvanted control group (*wk17 p* = 0.012* and wk20 p* = 0.0056) compared to the baseline (Fig. 6b, S.Fig. 11). However, no significant differences were observed between the two vaccination groups tested (Fig. 6b). This enhancement was further confirmed by the endpoint titre analysis (Fig. 6c). These findings may not be entirely surprising given that, often Gag-specific T cell responses have shown to correlate with Gag-specific IgG responses^15,23,25,41^.

### IL-4R antagonist adjuvanted vaccination showed an overall HIV-specific systemic and mucosal T cell bias compared to the unadjuvanted control

To obtain an overview of the total HIV-specific responses induced by the two vaccination strategies, total T cell responses (HIV Gag and Pol-specific polyfunctional and cytotoxic responses in different tissue compartments at wk20) and total B cell responses (Gag-specific IgG, Env-specific IgG, gp140 ADCC, NM neutralisation activity at wk20) were assessed using scatterplot quadrant analysis (T cell responses on the X axis, and B cell responses on the Y axis). Although the two vaccines contributed somewhat equally to the total B cell outcomes (*p* = 0.5623), IL-4R antagonist vaccination performed significantly better in the context of total T cell responses shown by the significant separation of the two vaccine groups on the x axis (Fig. 7a–d). Specifically, (1) where total systemic Gag-specific T cell responses showed *p* = 0.00065 (Fig. 7a); (2) addition of the HIV Pol-specific T cell responses into the analysis showed *p* = 0.0066 (Fig. 7b); and (3) total HIV Gag-specific systemic and mucosal poly-functional/cytotoxic T cell responses showed *p* = 0.0023 (Fig. 7c) (rectal only) and *p* = 0.01 with the addition of rectal and ILN at wk20 (Fig. 7d) compared to the unadjuvanted vaccine control.

## Discussion

Our findings revealed that the addition of a SOSIP HIV gp140 booster to the i.n. rFPV *gag pol env*/i.m. rMVA *gag pol* prime-boost vaccine strategy significantly bolstered the mucosal and systemic HIV Gag- and Pol-specific poly-functional/cytotoxic CD4^+^ and CD8^+^ T cell responses, and also induced some gp140-specific ADCC, p24gag- and gp140 Env-specific IgG, and weak neutralizing antibody responses to the common MN HIV strain. Overall, our i.n./i.m. vaccine strategy showed significant bias towards T cell than the B cell immunity. The performance of the IL-4R antagonist adjuvanted strategy in respect to Gag- and Pol-specific T cell immunity was superior to that of the unadjuvanted control. These findings further substantiated our recent SIV protective efficacy vaccine outcomes in outbred pigtail macaques, where the delivery sequence ‘route and vector’ (i.n. rFPV prime i.m. rMVA booster), was found to play a crucial role in the induction of highly poly-functional cytotoxic (CD107a^+^) mucosal and systemic HIV-specific CD4^+^ T cells, associated with protection^26^. Interestingly, in the current study much greater proportion of mucosal CD4^+^ T cells were also found to express granzyme B, K and TIA-1, unlike mucosal CD8^+^ T cells. We speculate that this may explain why a subset of macaques in the previous SIV challenge trial had detectable mucosal SIV-specific cytotoxic CD4^+^ T cells were protected against a high does intrarectal SIV_mac239_ challenge^26^.

The rFPV vaccines expressed HIV *gag/pol/gp140 env*, while the rMVA vaccines only expressed HIV *gag/pol* (no gp140 env), with the specific aim of firstly prime and boosting the Gag/Pol-specific T cell responses, followed by lagged expansion of the Env-specific antibody responses, post SOSIP HIV gp140 booster for optimal T and B cell immune outcomes (to avoid simultaneous expansion of Gag/Pol T cells as well as Env-specific antibody responses). Although the Env-specific humoral responses post i.m. SOSIP gp140 booster^34–36^ were anticipated, the continued rise of Gag- and/or Pol-specific poly-functional/cytotoxic CD4^+^/CD8^+^ T cell, and B cell responses throughout the study was unexpected. Interestingly, our previous SIV protective efficacy study also showed some Env-specific IgG antibody response following a single i.n. FPV *gag/pol gp140 env* prime, that was significantly enhanced immediately following the high does intra rectal SIV_mac239_ challenge^26^. SOSIP gp140 resembles the native form of the HIV virion, and mimic the structure of the virion-associated Env spikes, which is known to play a critical role in mediating virus entry to CD4^+^ T cells^35–37^.Thus, the unforeseen enhancement of the T cell and Gag-specific B cell responses post SOSIP gp140 booster, could likely be (1) a result of the ‘native-like trait’ of the SOSIP gp140 to serve as a CD4 receptor mediated agonistic-enhancement, bolstering the overall immune response and/or (2) due to our previously proposed ‘less is more’ theory (reduced antigen exposure may be more beneficial in inducing strong sustained protective immunity by preventing immune exhaustion)^26^, and/or (3) directly linked to mucosal/ systemic strategy used in this study. Thus, comprehensive evaluation of these unexpected/interesting mechanisms warrants further investigation.

Purely T cell based HIV vaccine approaches^2–4,42–44^ or purely antibody-based approaches^6–9,45^ have thus far yielded poor immune outcomes in human vaccine trials, as exemplified by the recent disappointing HVTN 702 phase IIb/III RV144 trial^9^. A body of evidence has shown the importance of cytotoxic HIV-specific T cells in controlling infection^46–50^, particularly HIV Gag-specific cytotoxic CD4^+^ and CD8^+^ T cells at the early stage of virus infection^51–53^. The immune mechanisms observed in HIV elite controllers emphasize the need to design novel HIV-vaccine strategies that can elicit such immune outcomes^49,54–58^. It is also now evident that an effective vaccine against a chronic mucosal pathogen such as HIV may need high quality cytotoxic mucosal and systemic T cell immunity for protection^26,59–61^. In the context prime-boost modalities, mucosal priming, has shown to induce high avidity HIV-specific mucosal T cell responses at the first line of defence, associated with protection, unlike systemic vaccination^12,13,41,62–65^. The current study, demonstrated the ability of intranasal rFPV prime to induce sustained poly-functional cytotoxic mucosal HIV Gag-specific CD4^+^ and CD8^+^ T cells not only at the local (lung) but also at the distal genito-rectal mucosae, which was further enriched by the co-expression of the IL-4R antagonist. Our study is consistent with previous work suggesting that the prime-boost modality route, timing, choice/order of the recombinant viral vectors and cytokine cell milieu/adjuvant, can all strongly influence the vaccine outcome^11–15,23,62,66^.

When unravelling the immune mechanisms linked to our vaccines, we have shown that IL-4R antagonist adjuvanted vaccine induce significantly reduced ILC2-derived IL-13 at the vaccination site 24 h post-delivery compared to the control^17^, responsible for the observed immune outcomes^24,26^. Recently, using range of viral vector-based vaccines we have also demonstrated that ILC-derived IL-13 is the master regulator of different ILC subsets, as well as DC activity at the early stage of vaccination, shaping the downstream adaptive immune outcomes^17–21,23^. Specifically, although IL-13 is known to be detrimental for high avidity T cell induction^13,15,23^, low IL-13 at the vaccination site has shown to be crucial for effective antibody differentiation/ formation^24,25^. Thus, the discrepancies observed in Env-specific antibody outcomes with the two vaccines, could most likely be associated with the level of IL-13 at the vaccination site (unadjuvanted > IL-4R antagonist). Interestingly, for decades different adjuvants have been used to manipulate DC activity to alter the adaptive immune outcomes^67–69^ We have recently found that not only the co-expressed adjuvants^17–19^ but adjuvants used with protein-based vaccines can significantly alter the ILC-derived IL-13 profiles at the vaccination site (S.Fig. 12), which subsequently modulate DC activity. In this study AddaVax was used with the SOSIP gp140 booster. However, taken together our recent findings^21,24,25^ (S.Fig. 12), we find that selecting an adjuvant that can induce much lower ILC2-derived IL-13 may prove useful in inducing more robust/improved Env-specific immune outcomes with our SOSIP gp140 booster strategy in a clinical setting. However, the role of the SOSIP gp140 trimer in modulating immune responses through binding and aggregating the CD4 receptor has not been evaluated as a boost antigen in previous viral vector primed animal vaccination studies.

This study also revealed that unlike in the mucosal compartment, Granzyme B and TIA expression was associated with systemic CD8^+^ T cells, whilst Granzyme K was mostly associated with systemic CD4^+^ T cells. Moreover, IL-4R antagonist adjuvanted vaccination induced significantly (1) elevated Granzyme B, K and TIA-1 triple positive cytotoxic CD4^+^ and CD8^+^ T cells in the systemic compartment, and (2) higher proportion of HIV Gag-specific systemic CD4^+^ T cells that expressed IL-2 (from wk13), and also Granzyme K, compared to the unadjuvanted control. The importance of IL-2 in maintenance of cytotoxic CD4^+^ T cells has been documented^70–72^. Interestingly, both vaccine strategies induced greater proportion of triple positive mucosal cytotoxic CD4^+^ cells (in all mucosal compartments tested specifically, rectal and ILN) compared to CD8^+^ T cells. An important role for cytotoxic CD4^+^ T cells in controlling HIV infection at the first line of defence^26–28,73–76^ as well as importance of mitigating cytotoxic CD8^+^ T cell escape have also been reported^77–81^. Thus, we propose that an approach such as IL-4R antagonist adjuvanted strategy together with SOSIP gp140 booster, which promotes elevated vaccine-specific cytotoxic mucosal CD4^+^ T cells, at the first line of defence the genito-rectal mucosae, whilst maintaining elevated cytotoxic CD4^+^ and CD8^+^ T cells in the blood compartment, may prove more useful in combating an infection such as HIV-1. Specifically, our strategy also has the ability to induce Gag- and Env-specific humoral immunity. However, prior to clinical evaluation, selecting the best commercial adjuvant that can pair with SOSIP-gp140 booster, (1) to promote the most robust Env-specific antibody outcome, possibly an adjuvant that induce low ILC2-derived IL-4/IL-13, and (2) the contribution of SOSIP gp140 antigen aggregating CD4 receptor to provide another adjuvant effect warrants investigation. Collectively, current findings indicate that this unique i.n./i.m. HIV IL-4R antagonist adjuvanted/SOSIP gp140 booster vaccination strategy offers promise for the future and warrants Phase 1 evaluation.

## Methods

### Animals and ethics statement

Twelve (12) healthy pigtail macaques (*Macaca nemestrina*) used in this study were randomly assigned into two groups. All animals were maintained, monitored daily, and experiments were performed in accordance with the Australian National Health and Medical Research Council (NHMRC) guidelines within the Australian Code of Practice for the Care and Use of Animals for Scientific Purposes. The animal ethics were approved by The Australian Animal Health Laboratory’s (AAHL), Animal Experimentation and Ethics Committee (AEEC). Protocol Number 1887. The pigtail macaques were obtained from the Australian macaque breeding facility and housed under PC3 conditions in large non-human primate cages (3 m × 2 m × 1 m) according to the NHMRC guidelines and protocols. All animals received standard primate feed and free access to water, maintained in a 12-h light/12-h dark cycle and cared daily by experienced animal technicians. All procedures, including immunisations and tissue harvest, were performed by experienced staff, a sedative, Ketamine (10 mg/kg intramuscular), was administered prior to performing any procedures. At the end of the trial period, macaques were ethically euthanized with Phenobarbitone.

### Vaccines

The FPV-HIV gag/pol parent vaccine was kindly provided by Dr. David Boyle and the AD8 env gp140 (B clade) gene was inserted into the rev site. For the FPV-HIV gag/pol/env IL-4R antagonist vaccine, the human gene for the IL-4 antagonist (IL-4C123) was also designed with a mutation at residue 123 and synthesized based on the human IL-4 sequence (in GenBank/NCBI Reference Sequence: NM_000589.2) as per described previously^26^. Similarly, MVA-HIV gag/pol (B clade) parent vaccine and MVA-HIV gag/pol IL-4R antagonist vaccine were also constructed as per described previously^26^. The vaccines were plaque purified and validated for insertion and expression of the human IL-4C123 and the HIV genes by PCR and western blotting using methods described previously^82,83^.

AD8 SOSIP gp140 was produced from an Env-expression plasmid^84^ derived from pNL(AD8)^85^ modified according to the “v4.1” stabilizing mutations^86^ and was expressed in Expi293F cells using the Expifectamine 293 Transfection system (Gibco) according to the manufacturer’s recommendations. Supernatants were harvested 3–5 days post transfection by pelleting cells at 10,000 × *g* for 30 min at 4 °C followed by filtration through a 0.22 µm filter. AD8 SOSIP gp140 was purified from harvested supernatant by immobilised metal affinity chromatography (IMAC) followed by size exclusion chromatography (SEC). Purified fractions were buffer exchanged into PBS and concentrated to 2 mg/ml using 100 kDa MWCO centrifugal filters, sterilised using 0.22 µm filters and stored at − 80 °C prior to use. Samples were verified endotoxin-free using the LAL Chromogenic Endotoxin Quantitation Kit (Pierce). The expression of gp140 Env and absence of p24 Gag proteins were confirmed by SDS PAGE Western blot analysis. Briefly, the proteins were separated based on size using 4–12% bis–tris NuPAGE gels (Invitrogen) with Precision Plus Protein Standards (Bio-Rad). Electrophoresed proteins were then transferred onto a polyvinylidene difluoride (PVDF) membrane and the membrane blocked using 5% (w/v) skim milk powder in PBST (0.05% (v/v) Tween in PBS). Blots were stained with either an anti-Env cocktail (2G12, F105, F240) or an anti-p24 antibody (71-31) at 1 µg/ml. These reagents were obtained through the NIH AIDS Reagent Program, Division of AIDS, NIAID, NIH: anti-p24 monoclonal antibody (mAb) 71-31^87^ from Dr. Susan Zolla-Pazner. anti-gp120 mAb 2G12 from Polymun, anti- gp120/gp41 mAbs F105^88^ and F240^89^ from Dr. Marshall Posner and Dr. Lisa Cavacini. Secondary staining using a goat anti-human IgG Fc HRP conjugate (Sigma) was performed and blots imaged using the Amersham Imager 600.

### Immunisation

Prior to immunization animals were administered with ketamine (10 mg/kg intramuscularly). Two groups of animals were primed intranasally using LMA MAD intranasal mucosal atomization device (Teleflex, USA), and directly to the lung using a tube placed into the trachea laryngoscopically with 2 × 10^8^ PFU of FPV-HIV or FPV-HIV-IL-4R antagonist vaccines respectively as per described previously^26^. Four weeks later, animals were boosted intramuscularly with 2 × 10^8^ PFU respective MVA-HIV or MVA-HIV-IL-4R antagonist vaccines as per described previously^26^. Eight weeks later, animals were boosted intramuscularly with SOSIP gp140 trimer 0.5 mg per macaque mixed with AddaVax (1:1) (InvivoGen, catalog # vac-adx-10).

### Sample collection

Animals were administered with ketamine (10 mg/kg intramuscularly) prior to performing any procedures. Whole blood samples were collected at various time points as per indicated in the timeline (Fig. 1a). Rectal biopsy and bronchoalveolar lavage were collected at week 5 (1-week post rMVA vaccination). At the end of the trial period (week 20), macaques were ethically euthanized with Phenobarbitone and rectal, cervical virginal, and lung tissues were collected.

### Mucosal tissue preparation

Following collection, all tissue samples (lung, rectal, cervical virginal tissue) were stored in complete RPMI-1640 (Sigma) containing 25% fetal calf serum (FCS, Gibco) at 4 °C and were processed the same day. Single cell suspensions were prepared as per described previously^23,26,41,65^. Briefly, samples were cut into small pieces and digested with complete RPMI containing 2 mg/ml collagenase (Sigma), 2.4 mg/ml dispase (Gibco), and 5 units/ml DNAse (Calbiochme La Jolla) for 45 min at 37 °C, vortexing every 15 min. Then digested samples were passed through sterile gauze and 100 um cell strainers (BD Falcon) to remove debris. Resulting cell suspensions were then treated with red blood cell lysis buffer containing 0.16 mM NH_4_Cl and 0.17 M Tris HCl (pH 7.65) for 2 min at room temperature. Cells were then washed three times with complete RPMI and passed through the cell strainers to remove any remaining debris. The final suspension was centrifuged and resuspended in 500 μl complete RPMI for counting. Iliac lymph nodes were cut into small pieces, directly mushed through cell strainers, washed with complete RPMI and then passed through cell strainers again to remove debris. The final suspension was centrifuged and resuspended in 1 ml complete RPMI for counting.

### Flow cytometry

Expression of cytokines (IFN-γ, TNF-α, IL-2) and cytotoxic markers (CD107a, Granzyme B, Granzyme K, and TIA-1) by CD4^+^ and CD8^+^ T cells from fresh whole blood and mucosal samples were assessed using flow cytometry as per described previously^26,90^. Basically, 235 μl whole blood was incubated with 1 μg/ml HIV 15 mer overlapping Gag and Pol peptide pools (NIH AIDS reagent program), or 1 μg/ml Staphyloccal Enteroxin B (SEB Sigma) together with 1 μg/ml co-stimulatory antibodies anti-CD28 (BD, clone L293) and anti-CD49d (BD, clone L25) at 37 °C for 6 h. Brefeldin A (BFA BioLegend) was added into each well after 1 h of initial stimulation. Cells were stained for surface markers of CD3 (Pacific Blue, BD, clone SP34-2), CD4 (PerCP, BD, clone L200), CD8 (Brilliant Violet 650, BD, clone SK1), and CD107a (APV-H7, BD, clone H4A3) in dark at room temperature for 30 min. Erythrocytes were lysed using BD FACS lysing solution in dark at room temperature for 10 min. Cells were permeabilized using BD Permeabilization solution in the dark, at room temperature for 10 min, washed using FACS wash buffer (1% FCS in sterile PBS) prior to intracellular staining. Anti-IFN-γ (APC, BD, clone B27), TNF (PE-Cy7, BD, clone Mab11), and IL-2 (FITC, BD, clone MQ1-17H12) were used to detect cytokine expression, and anti-granzyme B (APC, ThermoFisher, clone GB11), granzyme K (FITC, SCBT, clone GM6C3), and TIA-1 (PE, Beckman Coulter, clone 2G9A10F5) were used to evaluate cytotoxic markers expression. The cytotoxic marker staining set (Granzyme B, Granzyme K, and TIA-1) was not stimulated with HIV Gag/Pol peptide pool (as main aim was to monitor the total cytotoxic potential pre- and post-vaccination), the cytokine/CD107a and cytotoxic marker sets were stained separately in dark at room temperature for 40 min. Finally, cells were washed with PBS and fixed with BD stabilizing fixative. 1 × 10^6^ lymphocytes per sample were acquired on a BD LSRFortessa cytometer and data was analysed using FlowJo software v10 (https://www.flowjo.com/solutions/flowjo).

As for mucosal samples, cells were stimulated as per described above for 12 h at 37 °C with HIV 15 mer overlapping Gag peptide pool (NIH AIDS reagent program), then BFA was added and incubated for further 5 h. Note that due to limited cell yields no Pol-specific peptide stimulation was performed, Surface and intracellular staining were performed as per described previously^23,24,26,41^. Briefly, cells were washed twice with FACS buffer, and then surface stained with the same antibodies as above in the dark on ice for 30 min. Cells were fixed with IC-FIX (BioLegend) buffer, followed permeabilized with IC-Perm (BioLegend) buffer. Then, intracellular staining was performed in the dark on ice for 40 min. After intracellular staining, cells were washed and fixed with 0.5% paraformaldehyde (PFA). 500,000 cells were acquired on a BD LSRFortessa and data was analysed using FlowJo software v10 (https://www.flowjo.com/solutions/flowjo).

### Macaque FcγRIIIa dimer ELISA

The level of Env-specific antibodies that can cross-link dimeric macaque FcγRIIIa was assessed as previously described^91,92^. Briefly, ELISA plates were coated overnight with 50 ng/well of HIV-1_AD8_ gp140 SOSIP trimer or HIV-1_AD8_ gp120 monomer in PBS at 4 °C. Wells were then blocked with PBS containing 1% BSA and 1 mM EDTA for 1 h at 37 °C. Next, macaque plasma samples were added at a 1:25 dilution and incubated for 1 h at 37 °C. Biotinylated recombinant dimeric FcγRIIIa (*Macaca nemestrina*) was added at 0.1 µg/ml for 1 h at 37 °C, followed with HRP-conjugated streptavidin (1:10,000 dilution) for 1 h at 37 °C (Thermo Fisher Scientific). The wells were developed with TMB substrate (Sigma Aldrich) and the reaction was stopped with 1 M sulphuric acid. The absorbance values (450 nm) of the plasma samples were PBS-background subtracted and normalized to HIVIg (immune globulin pooled from plasma of HIV-1^+^ donors) (NIH-ARP).

### Detection of HIV gp140 Env-specific responses

To detect anti-Env IgG in NHP plasma, 96-well plates were first coated with a sheep anti-D7 mAb (D7324, Aalto Bio Reagents) in 100 mM NaCl and 20 mM Tris, pH 8.8 at a concentration of 2 μg/ml overnight at 4 °C. Plates were washed followed by blocking with 5% (w/v) skim milk powder in PBST for 1 h. AD8 SOSIP gp140 (D7 tagged) was loaded onto each plate at a concentration of 0.5 μg/ml and incubated for 3 h followed by washing. Serial dilutions of NHP plasma samples plus 1% normal sheep serum (NSS) were loaded and incubated for 2 h, then washed. Goat anti-human IgG Fc HRP conjugate (Sigma) was added at a concentration of 1:5000 plus 1% NSS and incubated for 1 h. Plates were washed and developed by the addition of KPL SureBlue TMB Microwell Peroxidase (Sera Care) and the absorbance measured at 450 nm with the background of 690 nm subtracted.

### Pseudovirus production and antibody neutralisation

HIV reporter virus pseudotyped with the selected Env was prepared by the co-transfection of an Env-deleted proviral plasmid (pDR-NLΔenv) with different HIV-1 envelope expression plasmids together with the plasmid containing into HEK-293T cells^33^. Virus stocks were produced with Env from HIV strains ADA, JRCSF and MN using Env expression plasmids (NIH Reagent program) to analyse the sera neutralisation activity. In addition, MuLV envelope from a non-related murine retrovirus was included as a non-specific infection control. Pseudoviruses were harvested 48 h after transfection and clarified by filtration through a 45 µm filter, aliquoted and stored at − 80 °C. Virus stocks were titrated in TZM-bl reporter cells (NIH Reference Program), and serum neutralisation activity was tested on 30,000 relative luciferase units (RLU) of pseudovirus. Duplicate samples of heat inactivated sera in 1: 2 serial dilutions was mixed with virus to 150 µl and incubated for 1 h at 37 °C. Before addition onto TZM-bl cells seeded in 96-well plates, DEAE Dextran (30 µg/ml) was added to the virus-antibody complex and incubated at 37 °C. After 72hrs luciferase activity was measured with Britelite Plus reagent (Perkin Elmer) and read on a FLUOStar microplate reader. A broad neutralising monoclonal antibody, B12 (NIH Reference Program) was included as positive control. The percentage of neutralisation was determined by calculating the difference in average RLU between virus control (incubated with DMEM) and test wells, dividing this result by the difference in average RLU between virus control and uninfected cell control well background. The percent neutralisation for each sera dilution was plotted and a non-linear regression model (log [Ab conc]. vs response/ 3 parameters curve) was used for a best fit neutralisation curve. The fitted curve was used to calculate Area Under the Curve (GraphPad) with 1 as the maximal value representing the AUC obtained for B12 and 0 for MULV.

### Detection of HIV-Gag specific IgG responses

HIV p24 Gag specific IgG antibodies were detected using ELISAs as per described previously^24–26,41^. Briefly, 96-well ELISA plates were coated with 5 ng/well of p24 Gag protein (HIV-1 B clade) in borate buffer (pH 8.5), overnight at 4 °C. Plates were washed and blocked with 5% skim milk in PBST (0.05% Tween 20 in sterile PBS) for 2 h at 37 °C. 50 μl of doubling diluted serum starting from 1/50 was added to each well and incubated at 37 °C for 6 h. Plates were then washed and incubated overnight at 4 °C with 50 μl HRP-conjugated anti-macaque IgG (HRP-IB3, NIH Non-human Primate reagent resource facility, University of Massachusetts Medical School) at 1/2500 dilution in 1% BSA/PBST. Finally, plates were washed and developed using peroxidase substrate and optical density (OD) was read at 450 nM on a Tecan Infinite m200 Pro Spectrometer.

### Data and statistical analysis

Statistical analysis was performed using GraphPad Prism v8 software (https://www.graphpad.com/scientific-software/prism/) or Microsoft Office 365 Excel. One-way ANOVA using Tukey’s multiple comparisons test and paired/unpaired T-test were used. The *p* values are denoted as: ns—*p* ≥ 0.05, *—*p* < 0.05, **—*p* < 0.01. ***—*p* < 0.001, ****—*p* < 0.0001. The percentage of total CD4^+^ or CD8^+^ T cells responding to HIV antigen (summation or add-up analysis) for each animal was calculated as (number of CD4^+^ or CD8^+^ T cells expressing IFN-γ^+^  + TNF^+^  + IL-2^+^  + CD107a^+^  + IFN-γ^+^TNF^+^  + IFN-γ^+^IL-2^+^  + TNF^+^IL-2^+^  + IFN-γ^+^TNF^+^IL-2^+^) / number of CD4^+^ or CD8^+^ T cells × 100%). Note that the single, double, or triple positive were calculated by avoiding any overlap (S.Fig. 13). The antibody data, the respective endpoint titres were calculated as serum dilution corresponding to, two times the average background OD reading of each animal at week zero. The endpoint titre value was applied to each sample with the highest dilution being recorded as the reciprocal of the dilution, plotted in log10 format. The summary scatterplot quadrant analysis was performed using R v3.6.3 (https://cran.r-project.org/bin/windows/base/old/3.6.3/), where total T cell responses for each animal was placed on the X axis and B cell responses on the Y axis. The T cell data consisted of the combination of the percentage of total CD4^+^ and CD8^+^ T cells responding to HIV from the add-up analysis together with add-up analysis of the cytotoxic marker expression (Granzyme B, Granzyme K and TIA-1). The B cell data consisted of the summation of Env- and Gag-specific IgG, ADCC, and MN neutralisation responses at the trial end point (wk20).

## Supplementary Information

Supplementary Information 1.

## Data Availability

The authors declare that all data supporting the findings of this study are available with the paper and supplementary files.
