# Supplementary material for: Mucosal IL-4R antagonist HIV vaccination with SOSIP-gp140 booster can induce high-quality cytotoxic CD4+/CD8+ T cells and humoral responses in macaques
Source: Sci Rep. 2020 Dec 16;10:22077. doi: 10.1038/s41598-020-79172-7 (PMC7744512; doi:10.1038/s41598-020-79172-7)
Supplement: Supplementary file 1 — Supplementary Information 1. [file 41598_2020_79172_MOESM1_ESM.pdf]

Mucosal IL-4R antagonist HIV vaccination with SOSIP-gp140 booster can induce high-quality cytotoxic CD4$^+$/CD8$^+$ T cells and humoral responses in macaques.

Z. Li[1], M. Khanna[1,2], S. L. Grimley[3], P. Ellenberg[3], C. A. Gonelli[3], Wen Shi Lee[3], T. H. Amarasena[3], A. D. Kelleher[4], D. F. J. Purcell[3], S. J. Kent[3 * †] and C. Ranasinghe[1* †]

[1]Molecular Mucosal Vaccine Immunology Group, Department of Immunology and Infectious Disease, The John Curtin School of Medical Research, The Australian National University, Canberra ACT 2601, Australia; [2]Department of Microbiology, Immunology and Parasitology, Louisiana State University Health Sciences Centre, New Orleans, LA 70112, USA; [3]Department of Microbiology and Immunology, Peter Doherty Institute, University of Melbourne, Melbourne VIC 3010, Australia; [4]Immunovirology and Pathogenesis Program, Kirby Institute, University of New South Wales, Sydney NSW 2052, Australia.

*Correspondence: Charani.Ranasinge@anu.edu.au; Telephone: +61 2 6125 4706 or skent@unimelb.edu.au.

[†]CR and SK contributed equally to this work.

Key words: HIV vaccine; prime-boost; rFPV; rMVA; IL-4R antagonist; SOSIP gp140; NHP; mucosal vaccination; poly-functional/cytotoxic CD4$^+$/CD8$^+$ T cell.

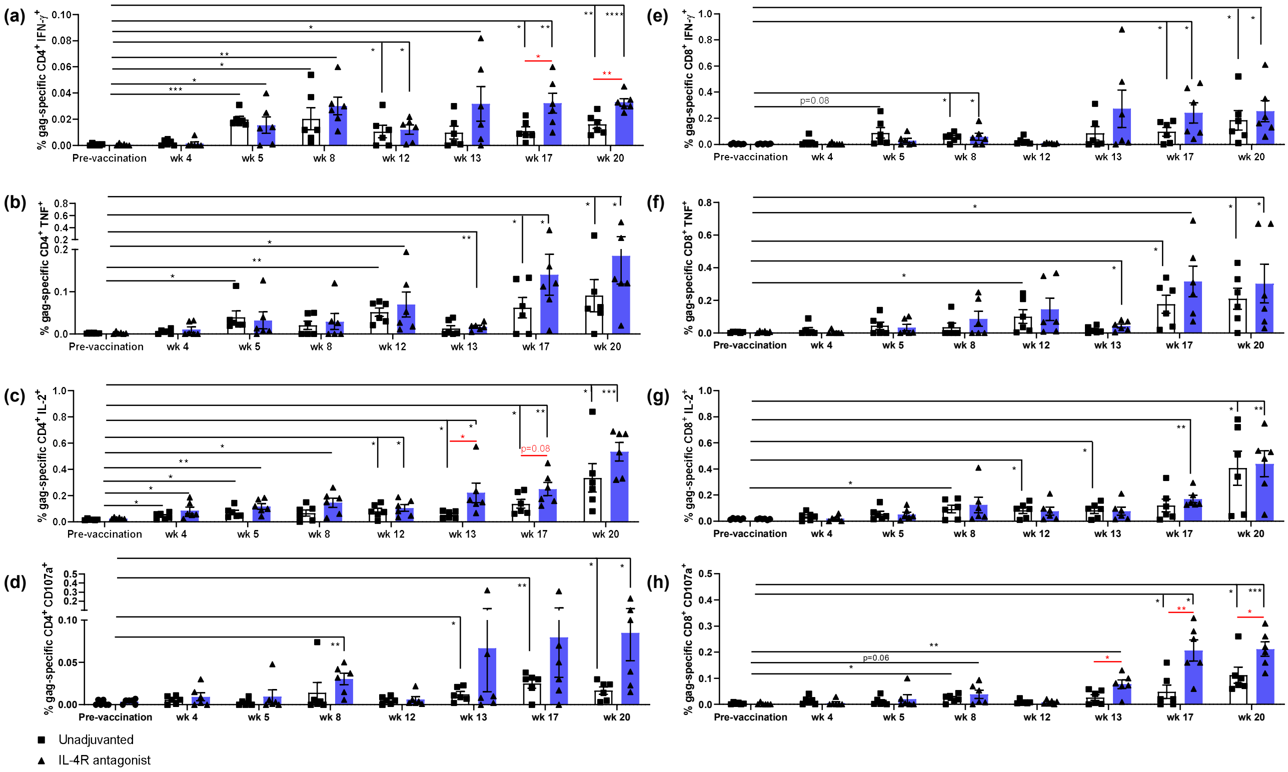

S. Fig. 1. Evaluation of HIV Gag-specific IFN-γ, TNF, IL-2, and CD107a expression by systemic CD4⁺ and CD8⁺ T cells.

Multi-colour flow cytometry analysis was performed to evaluate the HIV Gag-specific cytokine expression by CD4⁺ and CD8⁺ T cell as per described in Materials and Methods. The bar graphs represent **(a & e)** IFN-γ, **(b & f)** TNF, **(c & g)** IL-2, and **(d & h)** CD107a expression by HIV Gag-specific CD4⁺ and CD8⁺ T cells respectively, at all trial time points tested. Blue bars or lines represent IL-4R antagonist adjuvanted vaccinated group and white bars and black lines represent unadjuvanted vaccinated group.

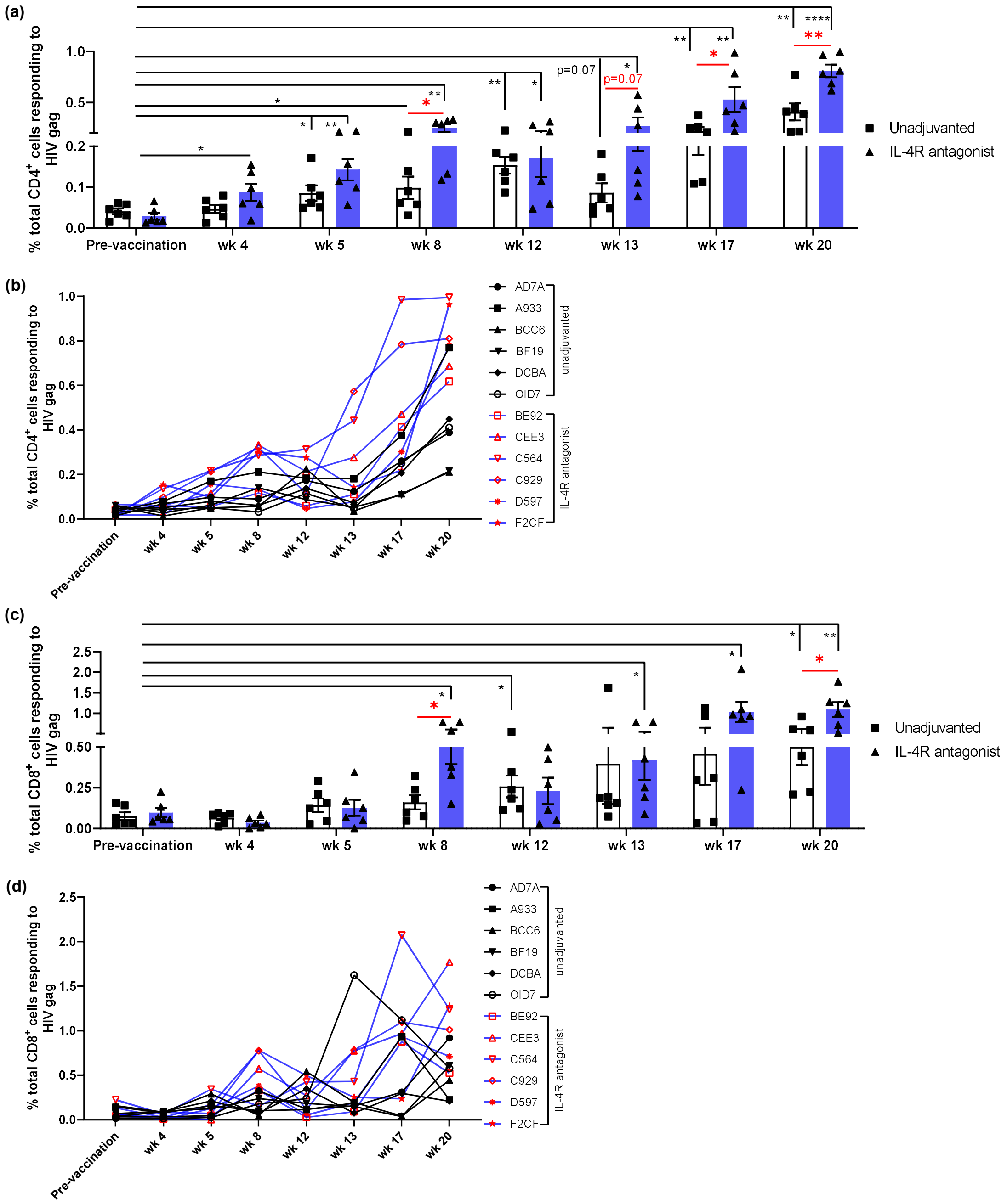

S. Fig. 2. Analysis of cytokine poly-functionality and summation (add-up) analysis of systemic HIV Gag-specific CD4⁺ and CD8⁺ T cells.

The poly-functional cytokine expression as well as cytokine and CD107a summation (add-up) analysis were performed to evaluate the overall HIV Gag-specific systemic CD4⁺ and CD8⁺ T cell responses in blood as per described in Materials and Methods. The bar charts represent total percentage of **(a)** CD4⁺ and **(c)** CD8⁺ T cells responding to HIV Gag, at all time points tested. The line graphs represent the trend of each animal **(b)** CD4⁺ and **(d)** CD8⁺ T cells responses. Blue bars or lines represent IL-4R antagonist adjuvanted vaccinated group and white bars and black lines represent unadjuvanted vaccinated group.

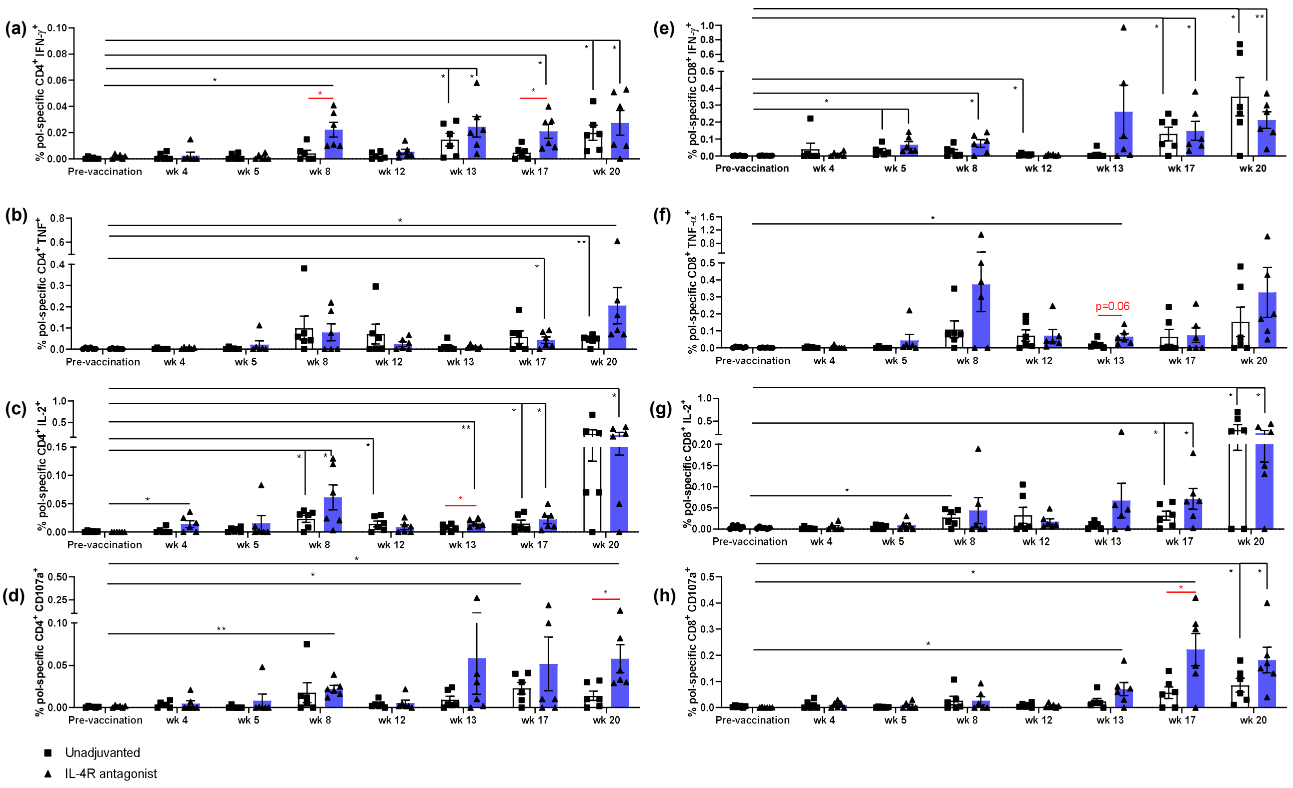

S. Fig. 3. Evaluation of HIV Pol-specific IFN-γ, TNF, IL-2, and CD107a expression by systemic CD4⁺ and CD8⁺ T cells.

Multi-colour flow cytometry analysis was also performed to evaluate the HIV Pol-specific cytokine expression by CD4⁺ and CD8⁺ T cell as per described in Materials and Methods. The bar graphs represent **(a & e)** IFN-γ, **(b & f)** TNF, **(c & g)** IL-2, and **(d & h)** CD107a expression by CD4⁺ and CD8⁺ T cells respectively, at all time points tested. Blue bars or lines represent IL-4R antagonist adjuvanted vaccinated group and white bars and black lines represent unadjuvanted vaccinated group.

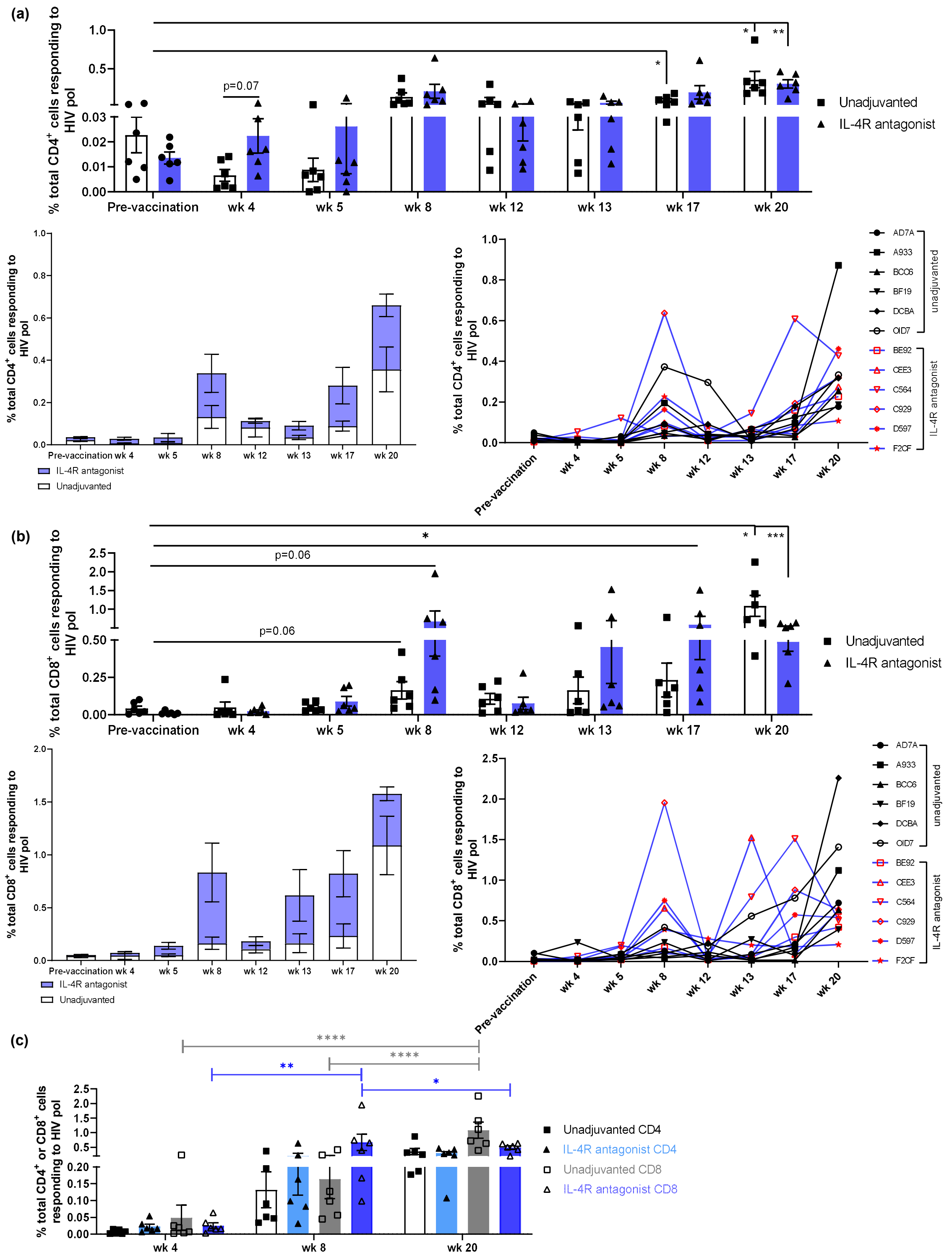

S. Fig. 4. Analysis of cytokine poly-functionality and summation (add-up) analysis of systemic HIV Pol-specific CD4⁺ and CD8⁺ T cells.

The poly-functional cytokine expression as well as cytokine and CD107a summation (add-up) analysis were to evaluate the overall HIV Gag-specific systemic CD4⁺ and CD8⁺ T cell responses in blood as per described in Materials and Methods. The bar charts represent total percentage of **(a)** CD4⁺ and **(b)** CD8⁺ T cells, responding to HIV Pol, at all time points tested. The line graphs represent the trend of each animal **(a)** CD4⁺ and **(b)** CD8⁺ T cells. The last bar graph **(c)** depict the comparison of HIV Pol-specific CD4⁺ and CD8⁺ T cells at three key time points (wk 4, wk 8, and wk 20). Blue bars or lines represent IL-4R antagonist adjuvanted vaccinated group and white bars and black lines represent unadjuvanted vaccinated group.

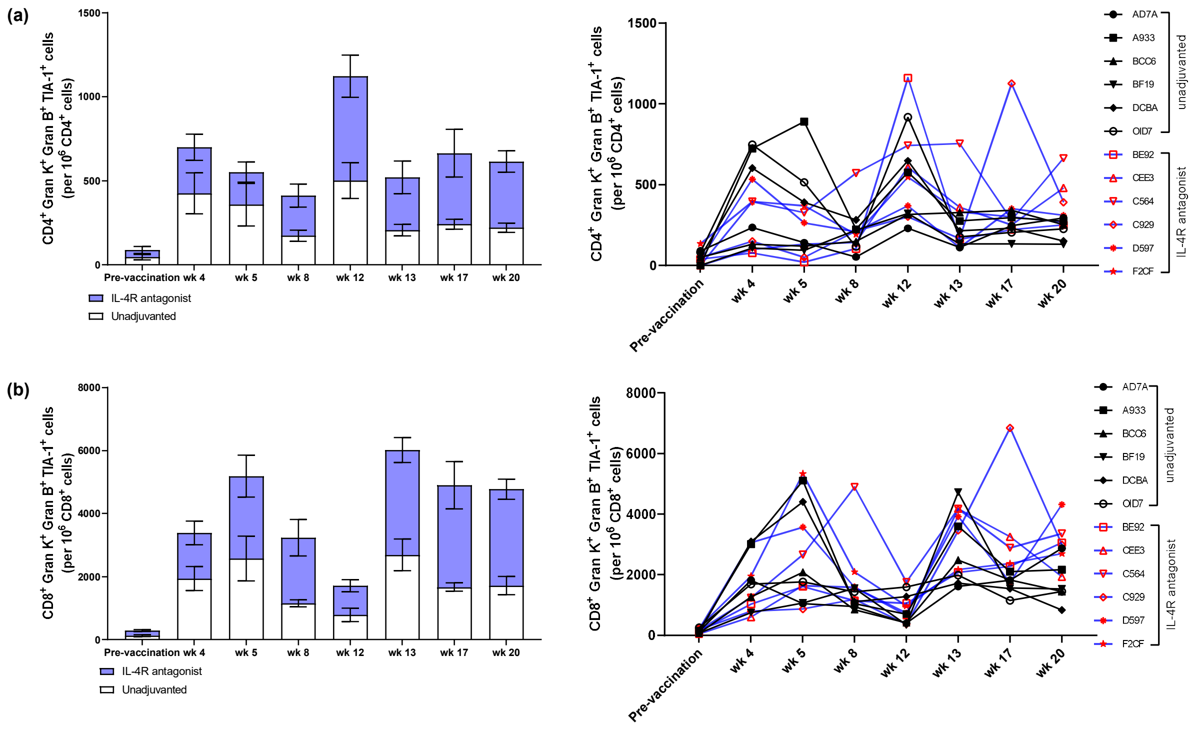

S. Fig. 5. Evaluation of CD4⁺ and CD8⁺ T cell that were triple positive for Granzyme K, Granzyme B, and TIA-1 in the blood compartment.

Data represent the number of CD4⁺ and CD8⁺ T cells that were triple positive for Granzyme K, Granzyme B, and TIA-1, back calculated to 1×10^6 cells. The stack bar charts represent number of **(a)** CD4⁺ and **(b)** CD8⁺ T cells expressing Granzyme K, Granzyme B, and TIA-1 at all time points tested. The line graphs represent the trend of each animal **(a)** CD4⁺ and **(b)** CD8⁺ T cells, respectively. Blue bars or lines represent IL-4R antagonist adjuvanted vaccinated group and white bars and black lines represent unadjuvanted vaccinated group.

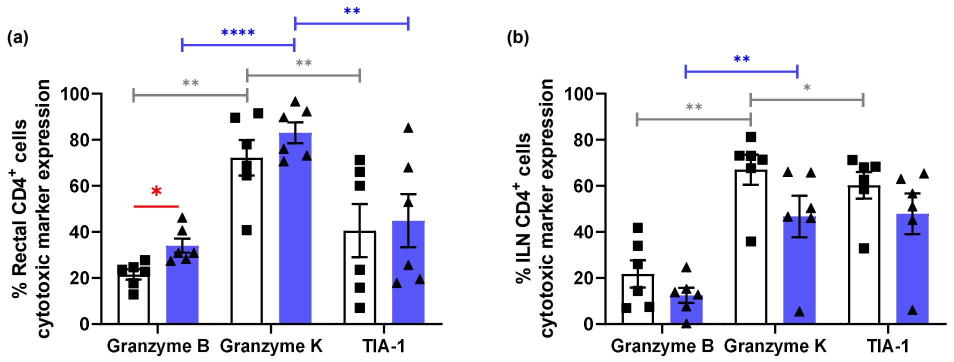

S. Fig. 6. Evaluation of Granzyme K, Granzyme B, and TIA-1 expression in rectal and ILN CD4$^+$ T cells.

The tissue samples were prepared as per described in Materials and Methods. The bar charts represent the percentage of CD4$^+$ T cells expressing Granzyme B, Granzyme K, and TIA-1 in **(a)** rectal and **(b)** ILN tissue compartments at 20 weeks. Blue bars represent IL-4R antagonist adjuvanted vaccinated group and white bars represent unadjuvanted vaccinated group.

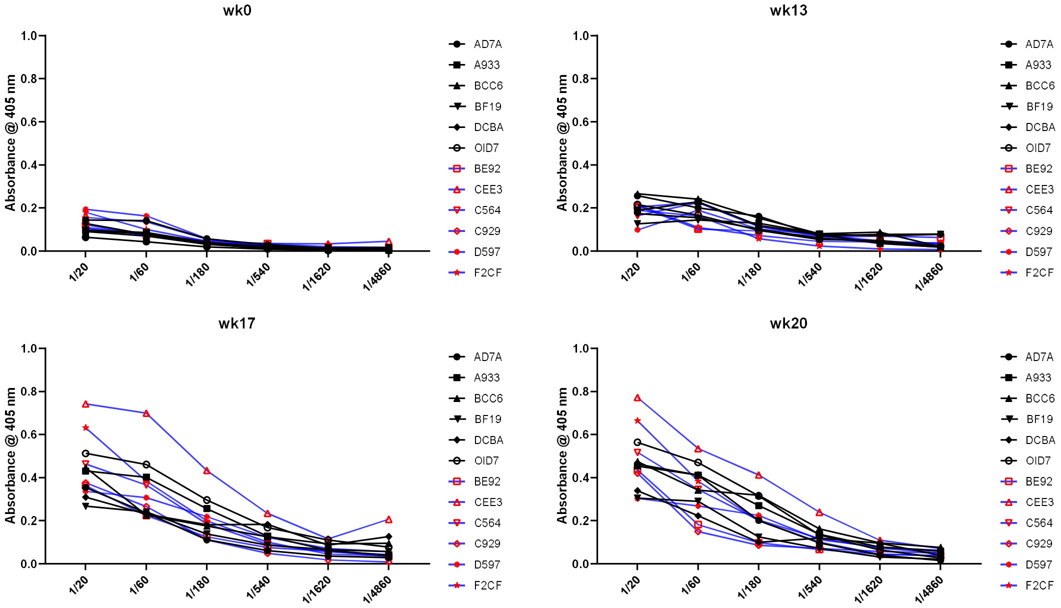

S. Fig. 7. Evaluation of HIV gp 140 Env-specific humoral responses.

HIV gp140 Env-specific total IgG were measured in the plasma using ELISA as per described in Materials and Methods. The line charts indicate the absorbance readings at 405 nm of plasma dilutions ranging from 1/20 to 1/4860 at pre-vaccination, wk 13, wk 17, and wk 20 time points. Blue lines represent IL-4R antagonist adjuvanted vaccinated group and black lines represent unadjuvanted vaccinated group.

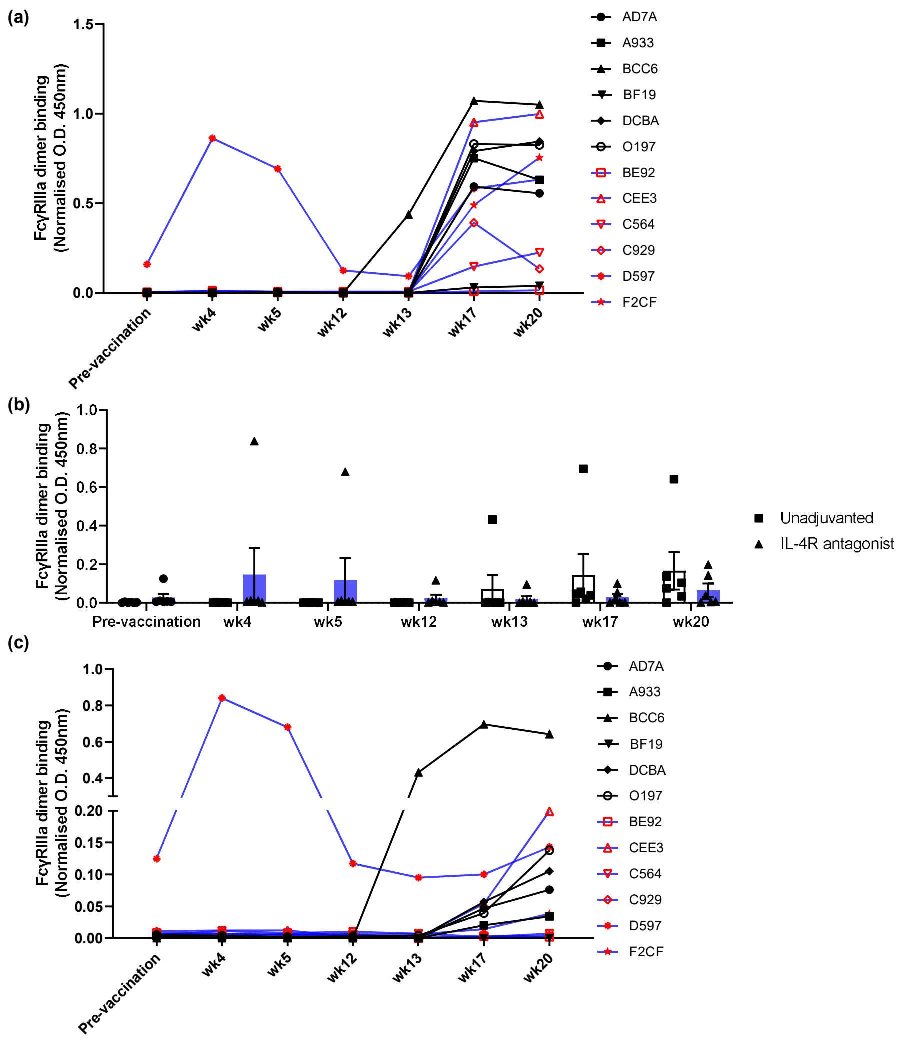

S. Fig. 8. Evaluation of HIV gp140 and gp120 ADCC activity.

HIV gp140 and gp120 ADCC activity were evaluated as per described in Materials and Methods. The line charts represent the ADCC activity trends of each animal measured against **(a)** gp140 and **(c)** gp120. The bar graph **(b)** represents the comparison of gp120-specific ADCC responses between the two IL-4R antagonist and unadjuvanted vaccination groups. Blue lines represent IL-4R antagonist adjuvanted vaccinated group and black lines represent unadjuvanted vaccinated group.

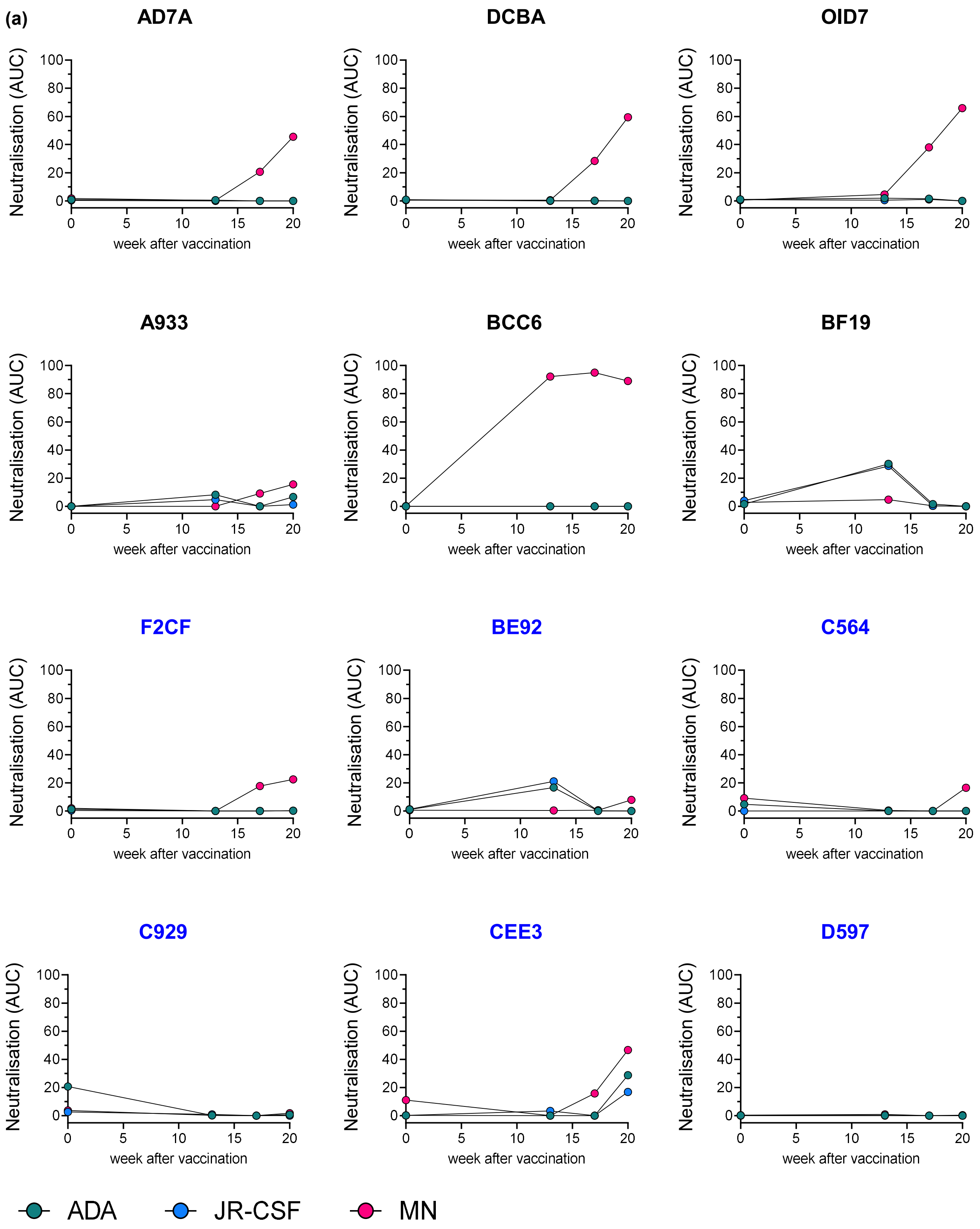
(a)
AD7A
DCBA
OID7
A933
BCC6
BF19
F2CF
BE92
C564
C929
CEE3
D597
Neutralisation (AUC)
week after vaccination
ADA
JR-CSF
MN

(b)

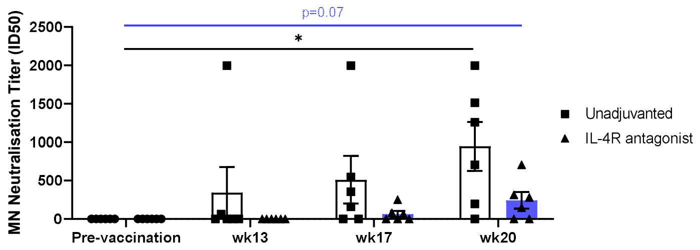

S. Fig. 9. Evaluation of HIV gp140 Env-specific neutralization antibody responses.

HIV gp140 Env-specific neutralization antibody responses were measured as per described in Materials and Methods. **(a)** The plots indicate neuralization antibody responses of different HIV strains in each animal at different time points (wk0, wk13, wk17, and wk20). The animals in blue indicate IL-4R antagonist and black indicate unadjuvanted control vaccination groups. **(b)** MN neutralisation endpoint titre at wk13, wk17 and wk20, compared to the pre-vaccination time point. The blue bars represent IL-4R antagonist adjuvanted and white bars represent unadjuvanted vaccination groups. The P-values are denoted as: ns - p≥0.05, * - p<0.05, ** - p<0.01. *** - p<0.001, **** - p<0.0001.

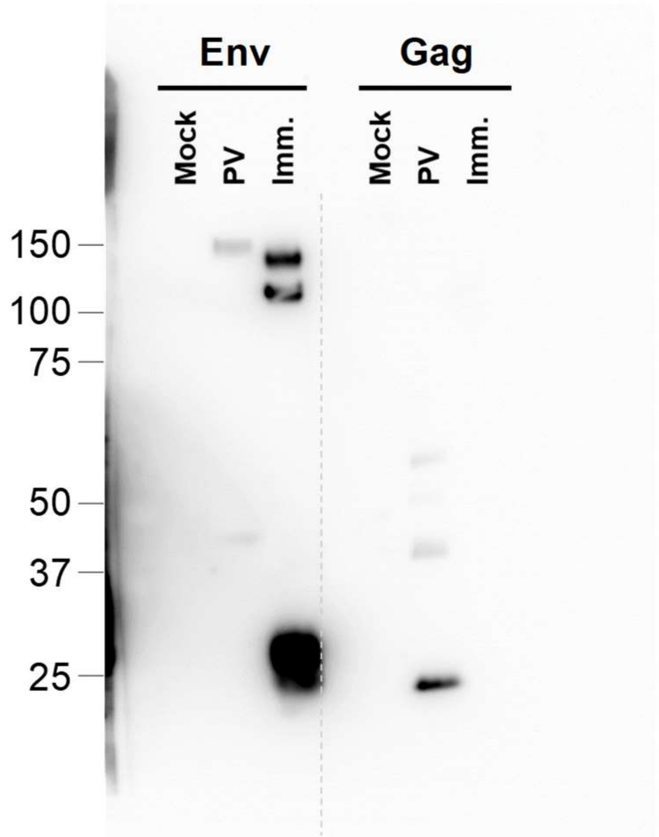

S. Fig. 10. Western blot analysis of the AD8 SOSIP gp140 Env and p24 Gag proteins (original full-length blot).

The gel was transferred to PVDF and the membrane was cut (indicated by the dashed line). The membranes were then probed with different mAbs and the two membranes were imaged together at the same time and with the same exposure.

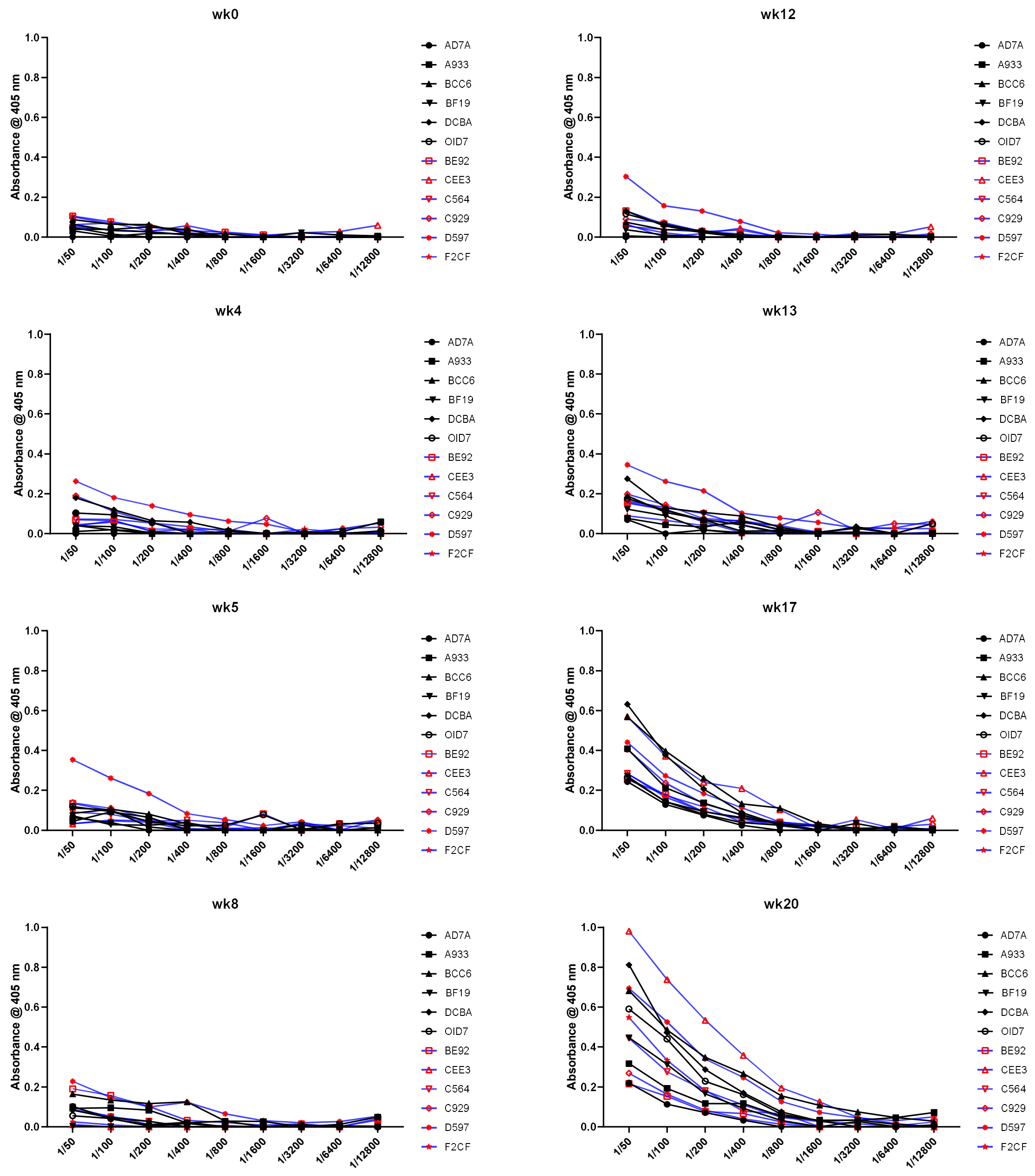

S. Fig. 11. Evaluation of HIV p24 Gag-specific antibody responses.

HIV p24 Gag-specific, total IgG levels were measured in plasma using ELISA as per described in Materials and Methods. The line charts represent absorbance readings at 405 nm for plasma dilutions ranging from 1/50 to 1/12800. Blue lines represent IL-4R antagonist adjuvanted vaccinated group and black lines represent unadjuvanted vaccinated group.

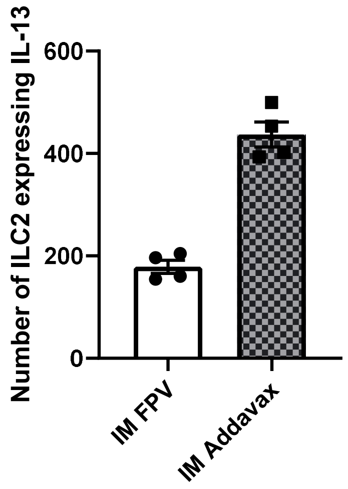

S. Fig. 12. Comparative analysis of ILC2-derived IL-13 in muscle 24h post rFPV and AddaVax (adjuvant only) vaccination.

Mice quadriceps muscle tissues were processed, and multi-colour flow cytometry analysis was performed to evaluate ILC2 IL-13 expression 24h post vaccination as per described previously [1,2]. ILC2 were gated as $CD45^{+}$ FSC^{low} SSC^{low} $Lineage^{-}$ $CD127^{+}$ $GATA3^{+}$ cells [3-5]. The graph represents number of IL-13 expressing ILC2 at the vaccination site 24h post vaccination. Note that rFPV vaccination showed very similar outcomes with the new and old flow cytometry gating strategies.

1 Li, Z., Jackson, R. J. & Ranasinghe, C. Vaccination route can significantly alter the innate lymphoid cell subsets: a feedback between IL-13 and IFN-γ. *npj Vaccines* **3**, 10, doi:10.1038/s41541-018-0048-6 (2018).

2 Li, Z., Jackson, R. J. & Ranasinghe, C. A hierarchical role of IL-25 in ILC development and function at the lung mucosae following viral-vector vaccination. *Vaccine: X* **2**, 100035, doi:https://doi.org/10.1016/j.jvacx.2019.100035 (2019).

3 Dutton, E. E. *et al.* Characterisation of innate lymphoid cell populations at different sites in mice with defective T cell immunity. *Wellcome Open Res* **2**, 117-117, doi:10.12688/wellcomeopenres.13199.3 (2017).

4 Romera-Hernández, M., Mathä, L., Steer, C. A., Ghaedi, M. & Takei, F. Identification of Group 2 Innate Lymphoid Cells in Mouse Lung, Liver, Small Intestine, Bone Marrow, and Mediastinal and Mesenteric Lymph Nodes. *Current Protocols in Immunology* **125**, e73, doi:10.1002/cpim.73 (2019).

5 Stehle, C., Hernández, D. C. & Romagnani, C. Innate lymphoid cells in lung infection and immunity. *Immunological Reviews* **286**, 102-119, doi:10.1111/imr.12712 (2018).

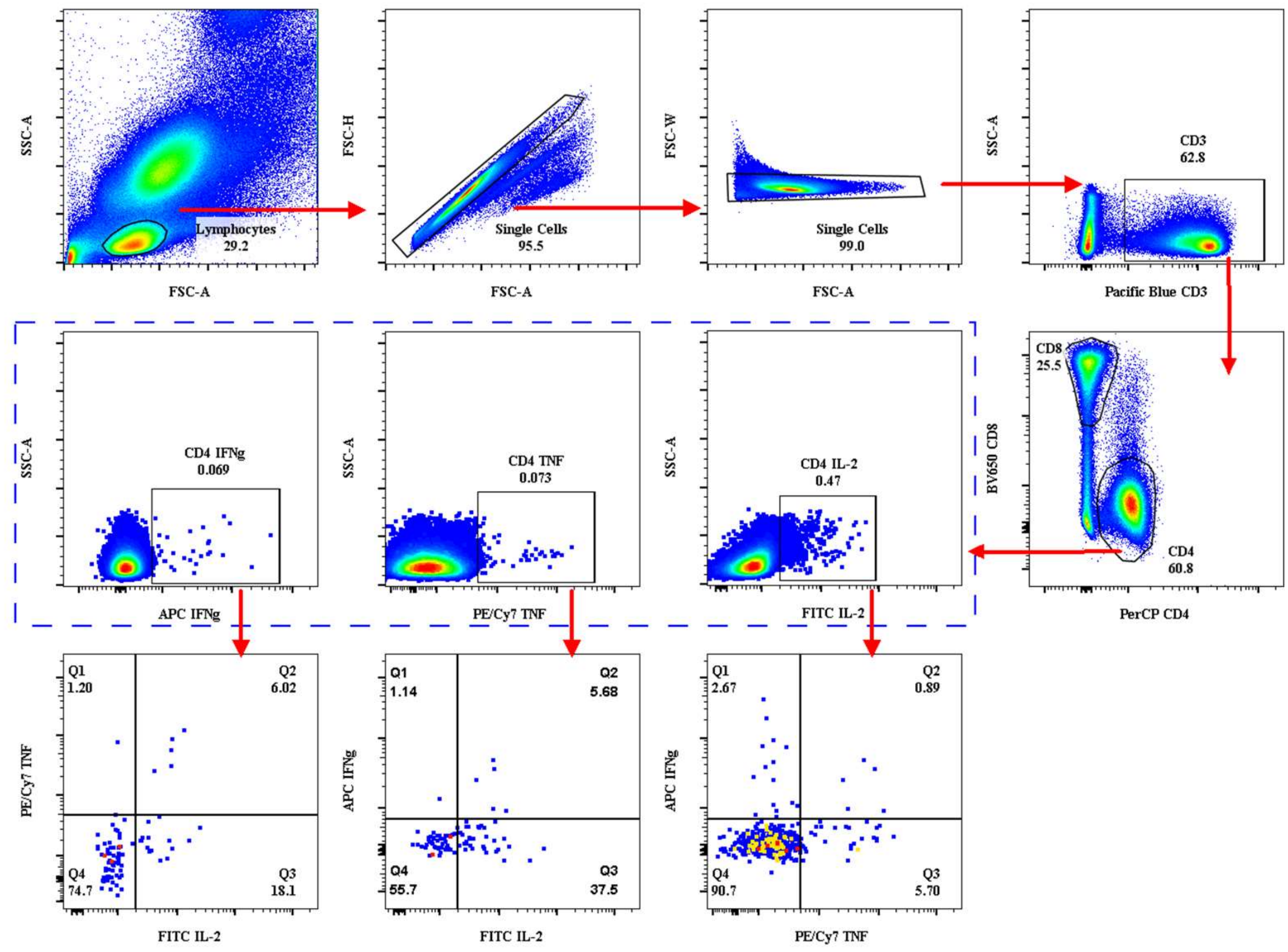

S. Fig. 13. Polyfunctional analysis gating strategy.

FACS staining was performed and data were acquired as per described in Materials and Methods. T cells were gated as CD3⁺ CD4⁺ or CD3⁺ CD8⁺ cells from total lymphocyte population after double discrimination. Next cytokine gating was performed for both CD4⁺ and CD8⁺ T cell subsets. For example, from CD4⁺ IFN-γ⁺ population, as per FlowJo analysis a quod gate was placed on the plot where IL-2 was on the X axis and TNF on the Y axis. The resulting four quadrants were defined as single positive (Q4: IFN-γ⁺ TNF⁻ IL-2⁻), double positive (Q1: IFN-γ⁺ TNF⁺ IL-2⁻, Q3: IFN-γ⁺ TNF⁻ IL-2⁺), and triple positive (Q2: IFN-γ⁺ TNF⁺ IL-2⁺) populations. Then, similar gating strategy was placed from TNF⁺ population with IL-2 on the X axis and IFN-γ on the Y axis, and the same was applied for IL-2⁺ population. This enabled us to cross check the poly-functional gating from different parent populations to make sure that all the gates were accurate. Also note that, all gates were placed based on negative and positive controls for each animal. This strategy enabled to effectively calculate the single, double, or triple positive populations without overlap, specifically when performing the add-up summation analysis.
